# Beyond Moore’s technologies: operation principles of a superconductor alternative

**DOI:** 10.3762/bjnano.8.269

**Published:** 2017-12-14

**Authors:** Igor I Soloviev, Nikolay V Klenov, Sergey V Bakurskiy, Mikhail Yu Kupriyanov, Alexander L Gudkov, Anatoli S Sidorenko

**Affiliations:** 1 Lomonosov Moscow State University, Skobeltsyn Institute of Nuclear Physics, 119991, Moscow, Russia; 2 Moscow Technological University (MIREA), 119454, Moscow, Russia; 3 All-Russian Research Institute of Automatics n.a. N.L. Dukhov (VNIIA), 127055, Moscow, Russia; 4 Solid State Physics Department, Kazan Federal University, 420008, Kazan, Russia; 5 Lukin Scientific Research Institute of Physical Problems, 124460, Zelenograd, Moscow, Russia; 6 Ghitu Institute of Electronic Engineering and Nanotechnologies ASM, Chisinau, Moldova

**Keywords:** energy-efficient computing, Josephson memory, superconducting computer, superconductor digital electronics, superconductor logics

## Abstract

The predictions of Moore’s law are considered by experts to be valid until 2020 giving rise to “post-Moore’s” technologies afterwards. Energy efficiency is one of the major challenges in high-performance computing that should be answered. Superconductor digital technology is a promising post-Moore’s alternative for the development of supercomputers. In this paper, we consider operation principles of an energy-efficient superconductor logic and memory circuits with a short retrospective review of their evolution. We analyze their shortcomings in respect to computer circuits design. Possible ways of further research are outlined.

## Introduction

Intel, one of the world’s largest chipmakers, “has signaled a slowing of Moore’s Law” [1]. The company has decided to increase the time between future generations of chips. “A technology roadmap for Moore’s Law maintained by an industry group, including the world’s largest chip makers, is being scrapped” [2]. Four years ago, Bob Colwell (former Intel chief IA-32 architect on the Pentium Pro, Pentium II, Pentium III, and Pentium IV) paraphrased the stagnation of semiconductor technology as follows [3]: “Officially Moore’s Law ends in 2020 at 7 nm, but nobody cares, because 11 nm isn’t any better than 14 nm, which was only marginally better than 22 nm” and “with Dennard scaling already dead since 2004, and thermal dissipation issues thoroughly constraining the integration density, the multicore era effectively ends, leading to the “dark silicon” problem, i.e., only parts of available cores can be run simultaneously”.

The mentioned fundamental changes are most clearly manifested in supercomputer industry. Energy efficiency becomes a crucial parameter constraining the advance of supercomputers [4–6]. The power consumption level of the most powerful modern supercomputer Sunway TaihuLight [7] is as high as 15.4 MW. It corresponds to a peak performance of 93 petaFLOPS (1 petaFLOPS is 10^15^ floating point operations per second). The power consumption level of the next generation exaFLOPS (10^18^ FLOPS) supercomputers is predicted [8] to be in the sub-gigawatts level. It is comparable to the power generated by a small powerplant and results in costs of hundreds of millions of dollars per year.

Following the corresponding roadmap [9], the goal for the power consumption level of exaFLOPS supercomputers should be of the order of ≈20 MW. This corresponds to an energy efficiency of 20 pJ/FLOP or 50 GFLOPS/W. Unfortunately, the energy efficiency of modern supercomputers is about one order smaller than required. For example, the energy efficiency [7] of the Sunway TaihuLight is 6 GFLOPS/W. It is understood that right after the need for space and the complex cooling infrastructure for exaFLOPS computers, energy efficiency is the next issue in high performance computing to be extraordinarily difficult, even if foreseeable advances in complementary metal-oxide-semiconductor (CMOS) technology are taken into account [10]. Low energy efficiency leads to high power consumption and also limits the clock frequency to 4–5 GHz. This frequency limit occurs due to “temperature” limitations posed to the integration level and switching rate of transistors. Note that cryogenic cooling of semiconductor chips will not solve the problem [11–12].

The future of high performance computing is most likely associated with one of alternative “Post-Moore’s” technologies where energy dissipation is drastically lower. It is expected that the future leading technology will be determined by 2030, while the period from 2020 to 2030 will be the “decade of diversity”. In this paper, we consider one of the most promising candidates for technological leadership: superconductor digital technology. The basic element switching energy here is of the order of 10^−19^ J, with no penalty for signal transfer. For a certain algorithm, superconducting circuits were shown to be up to seven orders of magnitude more energy efficient than their semiconductor counterparts, including the power required for cryogenic cooling [13]. The maturity level of superconductor technology can be illustrated by the notional prototype of a superconducting computer being developed under the IARPA programm “Cryogenic computing complexity” [14]. This is a 64-bit computing machine operating at 10 GHz clock frequency with a throughput of 10^13^ bit-op/s and an energy efficiency of 10^15^ bit-op/J at a temperature of 4 K. A prospective study shows that a superconductor computer could outperform its semiconductor counterparts by two orders of magnitude in energy efficiency, showing 250 GFLOPS/W [15].

The purpose of this paper is a review of the principles of operation of superconducting logic and memory circuits and an analysis of related issues with respect to the design of computer circuits. We certainly do not claim to be comprehensive and consider only the most common solutions. Our review contains two main parts describing logic and memory, correspondingly. In the first part we start with the examination of the physical basis underlying the operation of logic circuits. Superconductor logics are presented by two main branches: digital single flux quantum (SFQ) logics and adiabatic superconductor logic (ASL). The basic principles of SFQ circuit operation are shown for the example of the most popular rapid single flux quantum (RSFQ) logic. Its energy-efficient successors and competitors, low-voltage RSFQ (LV-RSFQ), energy-efficient RSFQ (ERSFQ), energy-efficient SFQ (eSFQ) and reciprocal quantum logic (RQL), are considered subsequently. ASL is described in the historical context of its development for ultra-energy-efficient reversible computation. The modern status is presented by two implementations of this logic. Superconducting adiabatic cells are used also in quantum computer circuits such as the ones fabricated by D-Wave Systems.

The second part of the review is devoted to cryogenic memory. Four approaches are described: SQUID-based memory, hybrid Josephson–CMOS memory, Josephson magnetic random access memory (JMRAM), and orthogonal spin transfer magnetic random access memory (OST-MRAM). They are presented in historical order of their development. At the end of each part of our review, we briefly discuss major challenges and directions of possible further research in the studied area.

## Review

### Logic

#### The physics underlying superconducting logic circuits

The fundamental physical phenomena underlying the operation of superconducting logic circuits are the superconductivity effects, the quantization of magnetic flux and the Josephson effect. The first one enables ballistic signal transfer not limited by a power necessary to charge the capacitance of interconnect lines. It provides the biggest advantage in energy efficiency in comparison to conventional CMOS technology. Indeed, superconducting microstrip lines are able to transfer picosecond waveforms without distortions with a speed approaching the speed of light, for distances well exceeding typical chip sizes, and with low crosstalk [16]. This is the basis for fast long-range interactions in superconducting circuits.

The absence of resistance (*R* = 0) leads to the absence of voltage (*V* = 0) in a superconducting circuit in stationary state. Superconducting current flow does not correspond to a difference of electrical potential (*V* = δ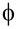) but to the difference of superconducting order parameter phases, δθ. The superconducting order parameter corresponds to the wave function of superconducting electrons |ψ|*e^iθ^* in the Ginzburg–Landau theory [17]. The magnetic flux Φ in a superconducting loop of inductance *L* provides an increase of the superconducting phase along the loop and results in a permanent circulating current *I* = Φ/*L*. This ratio is analogous to Ohm’s law *I* = *V*/*R*. It allows one to write linear Kirchoff equations for superconducting circuits.

The quantization of the magnetic flux introduces the fundamental difference between the operation of CMOS and of superconducting circuits. It follows from the uniqueness of the wave function of superconducting electrons. Indeed, the increase of the superconducting phase along a loop corresponds to the magnetic flux as 
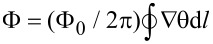
 (where Φ_0_ = *h*/2*e* ≈ 2 × 10^−15^ Wb is the magnetic flux quantum, *h* is the Planck constant, and *e* is the electron charge). This requires that 
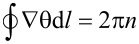
 (where *n* is an integer) and therefore Φ = *n*Φ_0_. Accordingly, the magnetic flux in a superconducting loop can take only values that are integer multiples of the flux quantum.

The physical representation of information is typically based on the quantization of the magnetic flux. For example, presence or absence of SFQ in a superconducting loop can be considered as a logical unity, “1”, or zero, “0”. Note that information is physically localized in such a representation. This is a fundamental difference compared to information representation in semiconductor circuits. The localization leads to a deep analogy between superconducting logic cells and von Neuman cellular automata [16] where short-range interactions are predominant.

The nonlinear element in superconducting circuits is the Josephson junction. It is a weak link between two superconductors, e.g., the most commonly used superconductor–isolator–superconductor (SIS) sandwich structure. One of the most important parameters of a Josephson junction is its critical current, *I*_c_. This is the maximum superconducting current capable of flowing through the junction. A Josephson junction can be switched from the superconducting to the resistive state by increasing the current above *I*_c_. The transition to the resistive state allows one to change the magnetic flux in a superconducting loop, and hence to perform a digital logic operation.

The dynamics of a SIS junction is commonly described in the frame of the resistively shunted junction model with capacitance (RSJC) [18]. This model presents a Josephson junction as a parallel connection of the junction itself transmitting only the superconducting current, *I*_s_, and a resistor and a capacitor with the corresponding currents, *I*_r_ = *V*/*R* and *I*_cap_ = *C*(∂*V*/∂*t*), where *t* is the time. The total current through the junction is the sum, *I* = *I*_s_ + *I*_r_ + *I*_cap_. This model is based on dc and ac Josephson effects that determine the superconducting current *I*_s_ and the voltage *V*.

The dc Josephson effect describes the superconducting current–phase relation (CPR). For SIS junctions it is *I*_s_ = *I*_c_ sin φ, where 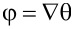 is the superconducting order parameter phase difference across the Josephson junction. It is called the Josephson phase. By presenting the relation between the superconducting order parameter phase and the magnetic flux as φ = 2πΦ/Φ_0_, we note that CPR couples current with the magnetic flux in a superconducting loop. The Josephson junction acts as a nonlinear inductance in the circuits, accordingly.

The ac Josephson effect binds the voltage at the Josephson junction in the resistive state with the superconducting phase evolution as *V* = (Φ_0_/2π)[∂φ/∂*t*]. According to this relation, an increase of the Josephson phase of 2π is accompanied by a voltage pulse across the junction such that ∫*V*d*t* = Φ_0_. Therefore, a single switching of the Josephson junction into the resistive state corresponds to the transmission of a SFQ pulse through the junction. The energy dissipated in the switching process is *E*_J_ ≈ *I*_c_Φ_0_ ≈ 2× 10^−19^ J, assuming a typical *I*_c_ ≈ 0.1 mA. The typical critical current value depends on the working (liquid helium) temperature, *T* = 4.2 K. For a proper operation of circuits it should be about three orders higher than the effective noise current value, *I*_T_ = (2π/Φ_0_)*k*_B_*T* ≈ 0.18 μA, where *k*_B_ is the Boltzmann constant.

The characteristic frequency of a Josephson junction switching process, ω_c_, is determined by the parameters of the Josephson junction, ω_c_ = (2π/Φ_0_)*I*_c_*R*_n_, where *I*_c_*R*_n_ is the characteristic voltage of the Josephson junction with *R*_n_ being the junction resistance in the normal state. Since SIS junctions possess a large capacitance, they are usually shunted by external resistors to avoid *LC* resonances. The resistance *R*_n_ is approximately equal to the resistance of the shunt, *R*_n_ ≈ *R*_s_, because *R*_s_ is much smaller than the tunnel junction resistance. For Nb-based junctions the characteristic frequency is of the order of ω_c_/2π ≈ 100–350 GHz (the characteristic voltage is about 0.2–0.7 mV). Superconducting digital circuits are predominantly based on tunnel junctions because of the high accuracy of their fabrication process and high characteristic frequencies.

**Figure 1:**
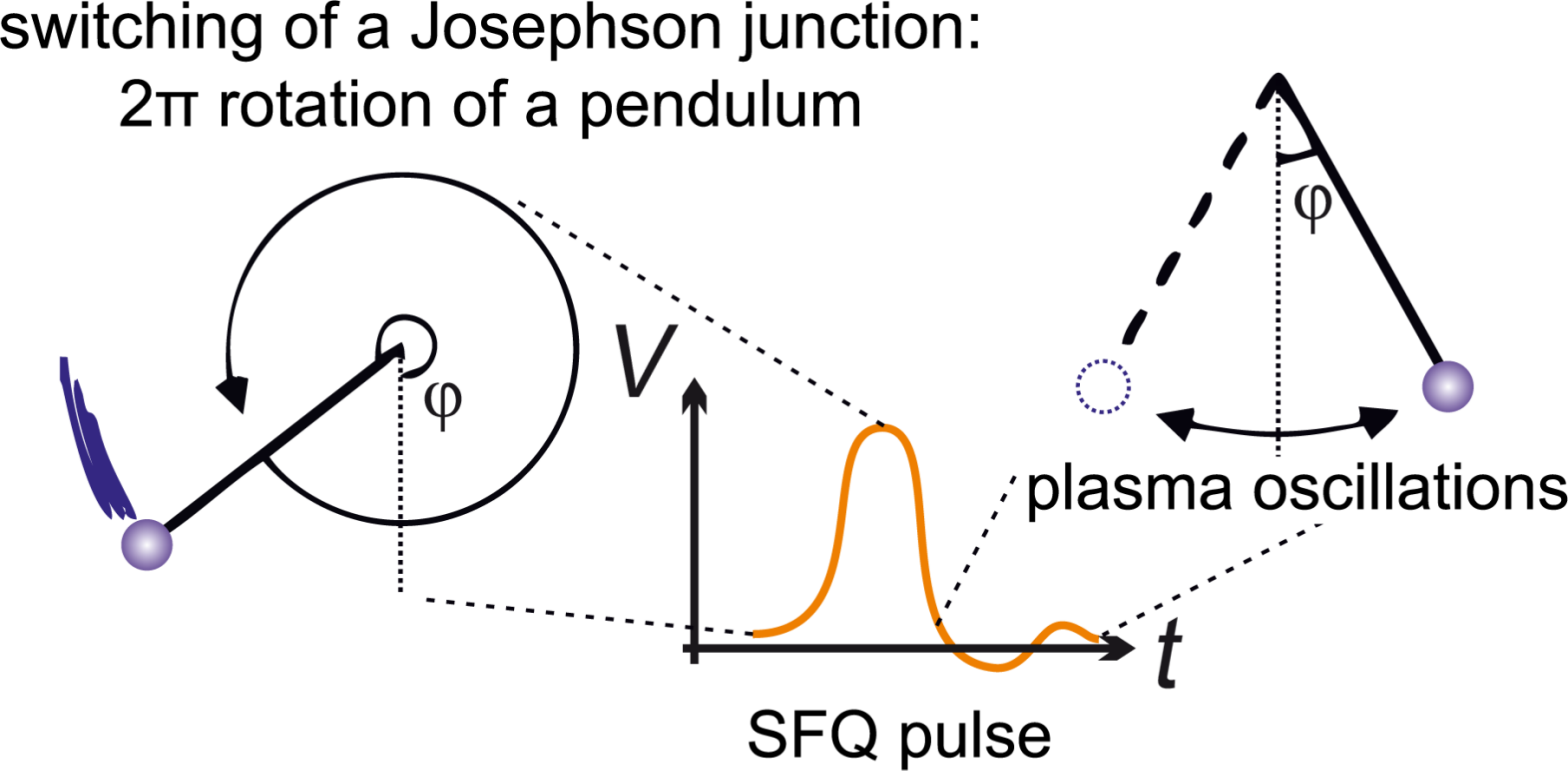
Voltage pulse on a Josephson junction corresponding to a SFQ transition and its mechanical analogy with the rotation of a pendulum.

By expressing the currents *I*_s_, *I*_r_ and *I*_cap_ of the RSJC model through the Josephson phase φ, we can present the total current flowing through the junction in the following form:

[1]
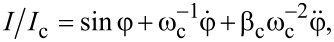


where β_c_ = ω_c_*R*_n_*C* is the Stewart–McCumber parameter reflecting the impact of the capacitance, and the dots denote the derivative with respect to time. Equation 1 is quite analogous to the one for a mechanical pendulum with the moment of inertia 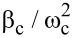 (capacitance here is analogous to mass), the viscosity factor 1/ω_c_ (resistance determines damping), and the applied torque *I*/*I*_c_. This simple analogy allows to consider a superconducting digital circuit as a net of coupled pendulums. A 2π rotation of the pendulum is accompanied by subsequent oscillations around a stable equilibrium point (Figure 1). In Josephson junction dynamics they are called “plasma oscillations”. The plasma oscillation frequency is ω_p_ = 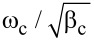 = 
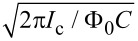
. For a proper operation of a logic cell, these oscillations should vanish before subsequent switching of the Josephson junction. Compliance with this requirement can be achieved through β_c_ ≈ 1, ω_p_ ≈ ω_c_. The clock frequency is accordingly less than ω_c_, and is under 100 GHz in practical circuits.

The complexity of a superconducting circuit realizable on a chip is determined by the dimensions of a Josephson junction. The area of a Josephson junction is closely related to its critical current density, *j*_c_. This parameter is one of the most important in the standard Nb-based tunnel junction fabrication process. It is fixed by materials properties of the insulating interlayer Al_2_O_3_ between the superconducting Nb electrodes, and its thickness *d* ≈ 1 nm. The critical current density value lies typically in the range *j*_c_ = 10–100 μA/μm^2^. The corresponding specific capacitance of the Josephson junction is *c* ≈ 40–60 fF/μm^2^. A variation of the critical current of a Josephson junction, *I*_c_ = *aj*_c_, is obtained by a variation of its area, *a*. It is accompanied by a variation of the capacitance of the Josephson junction, *C* = *ac*. The shunt resistance is adjusted, in accordance with the condition β_c_ = 1, as 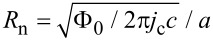. Its area is defined by the area of the Josephson junction, *a*, the minimum wiring feature size [19–20] (ca. 0.5–1 μm), and the sheet resistance of the used material (2–6 Ω per square for Mo or MoN*_x_*) [19–20].

While the weak link area of the Josephson junction itself is typically *a* ≈ 1 μm^2^ for *j*_c_ = 100 μA/μm^2^, its total area with the shunt is larger by an order of magnitude. The corresponding available density of Josephson junctions on a chip is 10^7^/cm^2^. The complexity of superconducting circuits becomes limited to 2.5 million junctions per square centimeter under the assumption that only a quarter of the chip area can be occupied by Josephson junctions (with taking interconnects into account) [19]. The circuits can be further expanded using multi-chip module (MCM) technology [21–22].

#### Digital single flux quantum logic

**Figure 2:**
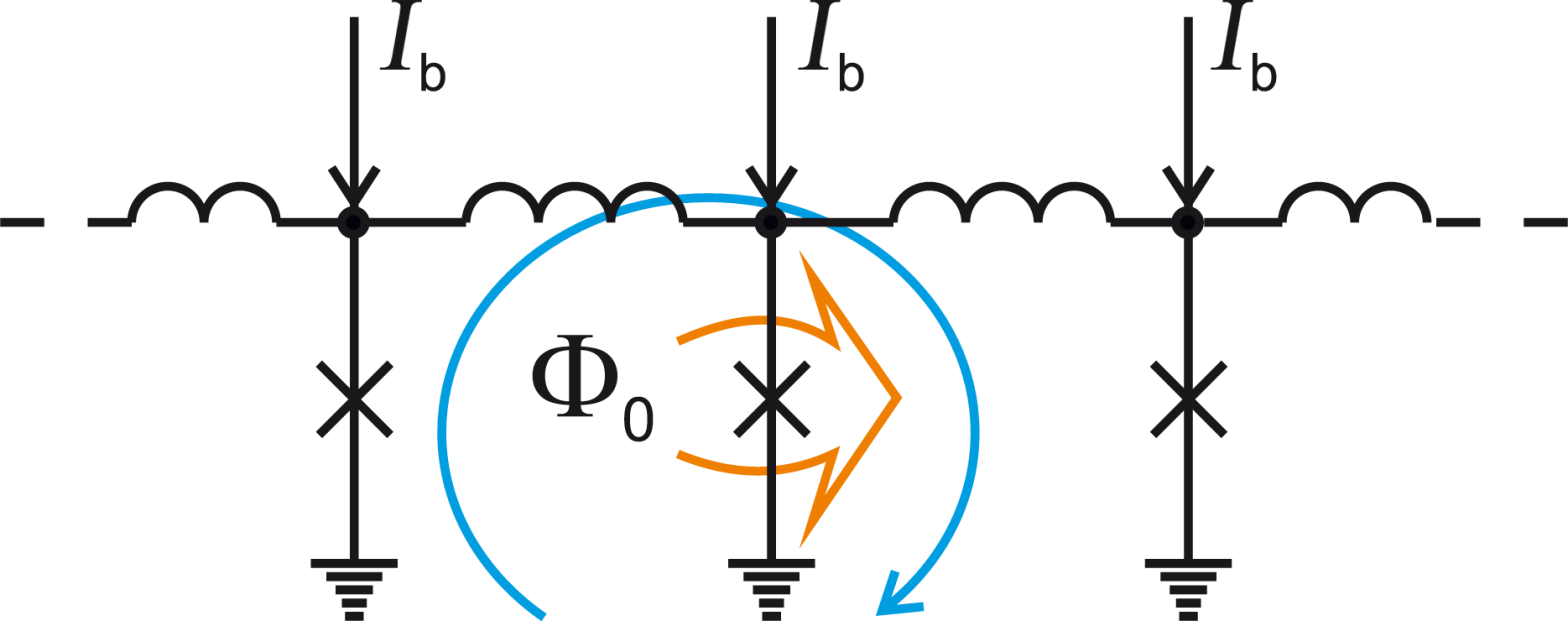
Josephson transmission line. Josephson junctions are shown by crosses. *I*_b_ is the applied bias current. The blue arrow presents an SFQ circulating current. The orange arrow highlights the Josephson junction in the resistive state.

**Basic principles of operation of SFQ circuits:** Data processing in SFQ circuits can be discussed using an example of RSFQ cell operation. An RSFQ data bus is shown in Figure 2. It is a parallel array of superconducting loops composed of Josephson junctions (shown by crosses) and superconducting inductances. This structure is called the Josephson transmission line (JTL). SFQ can be transferred along this JTL by successive switchings of Josephson junctions. The switching is obtained by summing the SFQ circulating current and the applied bias current *I*_b_. The transition of a Josephson junction into the resistive state leads to a redistribution of the SFQ circulating current toward the next junction. The redistribution process ends by the next junction switching and the successive return of the current junction into the superconducting state.

This example shows the basic principle of operation of SFQ logic cells. It reduces to the summation of currents, which are SFQs currents and bias currents. This summation leads (or does not lead) to successive switching of Josephson junctions resulting in the reproduction (or not) of SFQs. In the RSFQ convention [16,23], the arrival of an SFQ pulse during a clock period to a logic cell has the meaning of a binary “1”, while the absence of the SFQ pulse means “0”.

**Figure 3:**
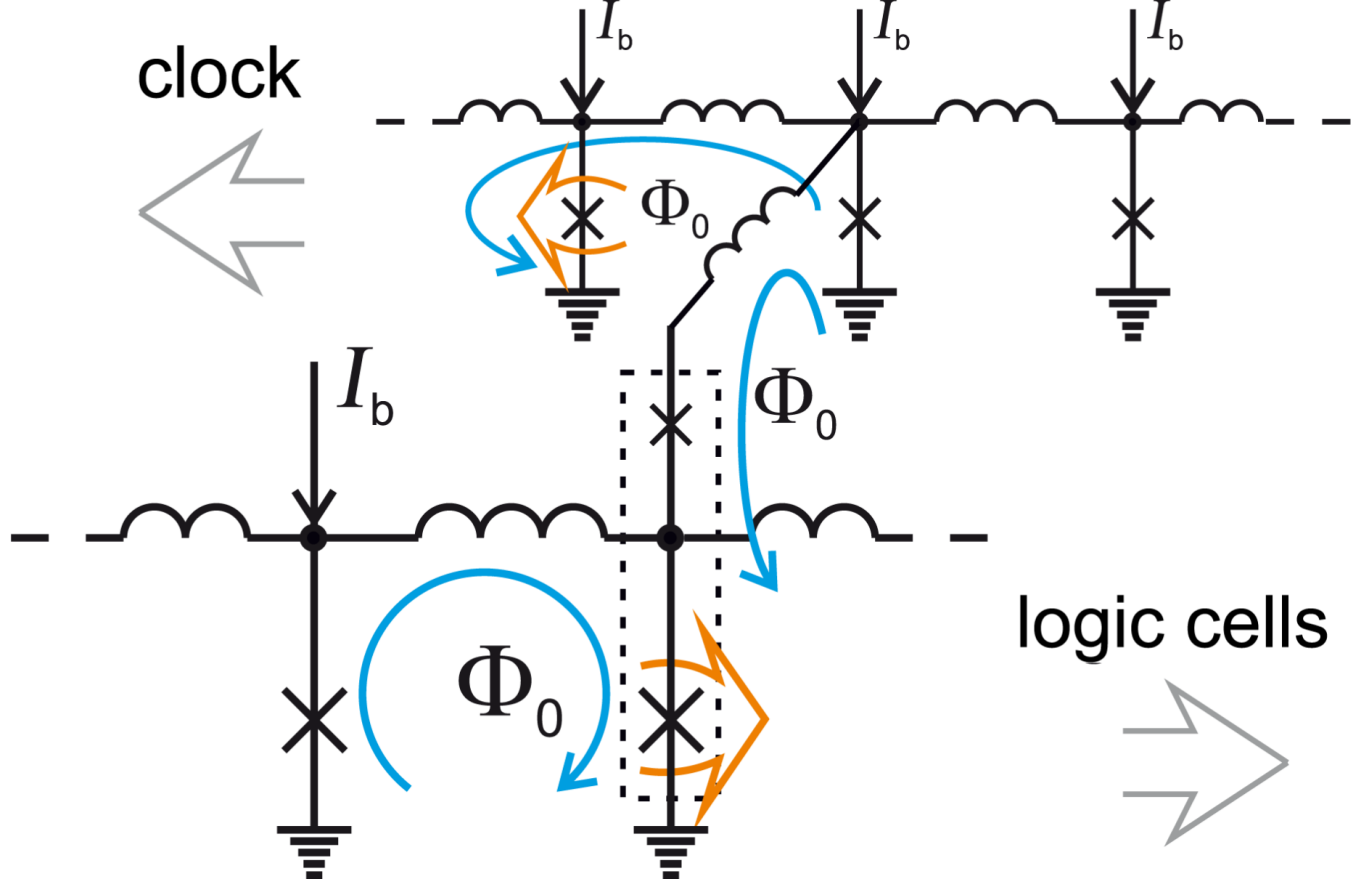
An RSFQ logic cell coupled to a clocking JTL. *I*_b_ is the applied bias current. Blue arrows present circulating currents of SFQs. Orange arrows highlight Josephson junctions in the resistive state. The dotted rectangle marks the decision-making pair.

Figure 3 illustrates an example of clocked readout of information from an RSFQ logic cell. Clocking is performed by means of SFQ application to the cell. The upper JTL in Figure 3 is used for SFQ clock distribution. SFQs are allotted to the cell through extra branch coupled to the JTL as shown. Note that the Josephson junction clones SFQs at the branch point. Readout operation is performed by a couple of junctions marked by the dotted rectangle. This couple is commonly called the decision-making pair. The existence (or absence) of an SFQ circulating current in the logic cell loop makes the lower junction to be closer to (or farther from) its critical current compared to the upper junction. The clocking SFQ switches the lower (or upper) junction, correspondingly. SFQ reproduction by the lower junction means a logical “1” to the output, while the absence of the SFQ means a logical “0”.

One can see a couple of typical features of SFQ circuits in the presented example. The logic cell acts as a finite-state machine. Its output depends on a history of its input. This particular cell operates as a widely used D flip-flop (“D” means “data” or “delay”), which are the basis of shift registers. Note that its realization is much simpler than that of the semiconductor counterparts. RSFQ basic cells are flip-flops, and therefore RSFQ logic is sequential logic. This is in contrast with semiconductor logic which is combinational one (where the output of a logic cell is a function of its present input only). Since only one clocked operation is performed during a clock period (some operations can be performed asynchronously), a processing stage in RSFQ circuits is reduced to a few logic cells. This is also completely opposite to conventional semiconductor circuits.

**RSFQ logic:** RSFQ logic dominates in superconductor digital technology since the 1990s [24]. Many digital and mixed-signal devices such as analog-to-digital converters [25–26], digital signal and data processors [27] were realized on its basis. Unfortunately, energy efficiency did not matter in the days of RSFQ development. The high clock frequency was thought to be the major RSFQ advantage in the beginning. An extremely fast RSFQ-based digital frequency divider [28] (T flip-flop) was presented just about a decade after the invention of RSFQ logic. Its clock frequency was as high as 770 GHz. It is still among the fastest ever digital circuits.

**Figure 4:**
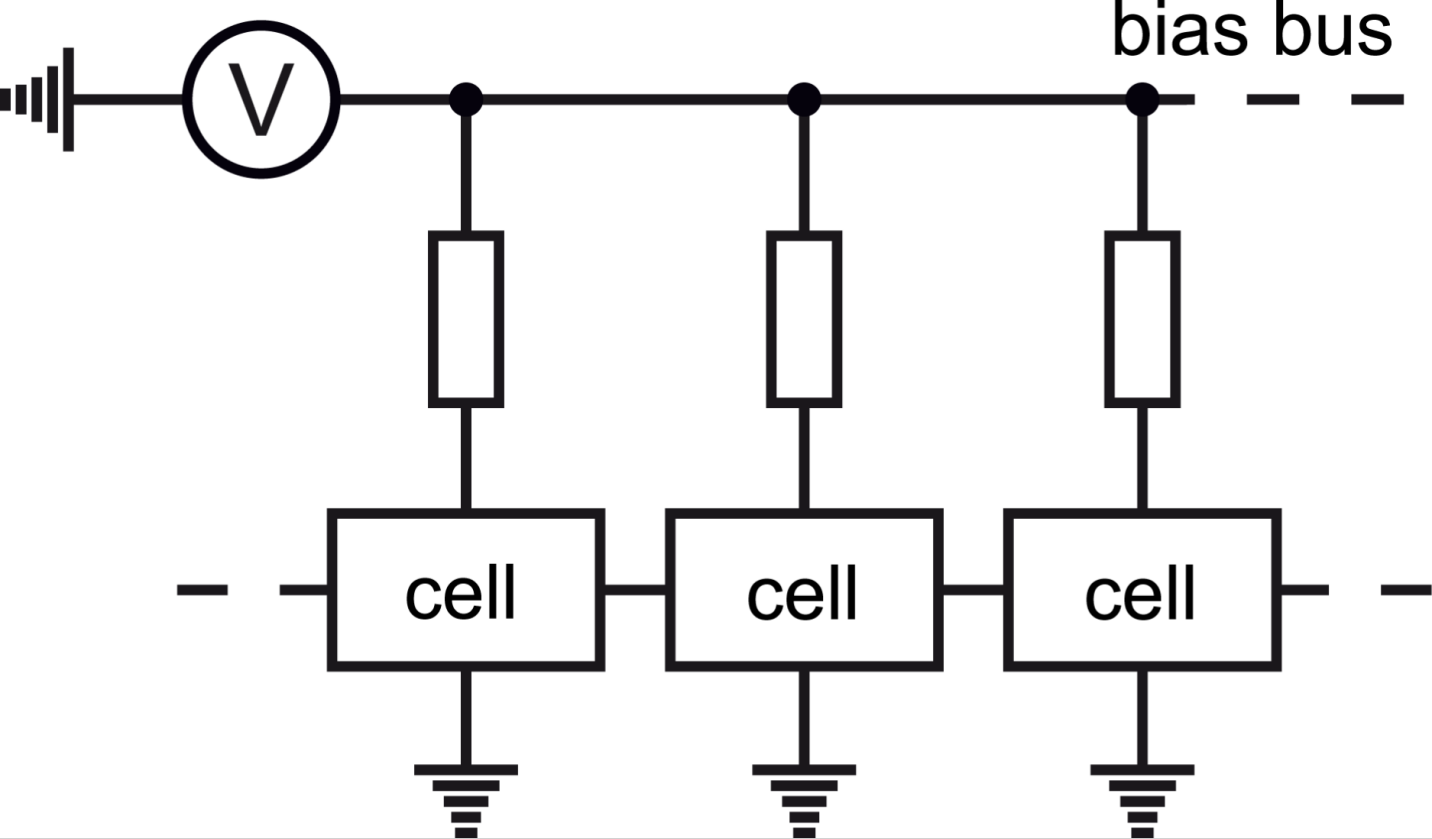
RSFQ power supply scheme.

The first RSFQ basic cells were superconducting loops with two Josephson junctions (commonly known as the superconducting quantum interference devices, SQUIDs). These cells were connected by resistors [23,29] (so “R” stood for “resistive” in the abbreviation). Power supply bus coupling was also resistive. While resistors connecting the cells were rather quickly substituted for superconducting inductances and Josephson junctions [30], the ones in feed lines remained until recent years, see Figure 4. They determined the stationary power dissipation, *P*_S_ = *I*_b_*V*_b_, where *I*_b_ and *V*_b_ are the dc bias current and according voltage. The bias current is typically *I*_b_ ≈ 0.75*I*_c_. The bias voltage has to be higher than the characteristic voltage of the Josephson junction by an order of magnitude, *V*_b_ ≈ 10 × *I*_c_*R*_n_, to prevent the redistribution of the bias current. This requirement determined the bias resistors values. The typical stationary power dissipation of an RSFQ cell [11] is *P*_S_ ≈ 800 nW.

Another mechanism leading to power dissipation is the switching of Josephson junctions. This dynamic power dissipation is defined as *P*_D_ = *I*_b_Φ_0_*f*, where *f* is the clock frequency. For a typical clock frequency of 20 GHz, *P*_D_ is at the level [11] of ca. 13 nW. This means, the dynamic power dissipation is about 60 times less than the stationary dissipation. Hence, the main efforts to increase the energy efficiency of RSFQ circuits were aimed at a decrease of the stationary energy dissipation. The energy-efficient successors of RSFQ, i.e., LV-RSFQ, ERSFQ and eSFQ, are presented below.

**Low-voltage-RSFQ:** The first step toward the reduction of *P*_S_ was the decreasing of the bias voltage. Bias current redistribution between neighboring cells in low-voltage RSFQ (LV-RSFQ) is damped by the introduction of inductances connected in series with the bias resistors in feed lines [31–35]. Unfortunately, this approach limits the clock frequency. Indeed, an increase of the clock frequency is accompanied by an increase of the average voltage 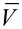 across a cell (according to the ac Josephson effect). This in turn leads to a bias current decrease proportional to 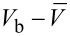. The latter finally results in the malfunction of the cell [36]. This tradeoff and the requirement of an additional circuit area for inductances in the feed lines practically limit the application of this approach. Since the static power dissipation is not eliminated, this is a somewhat half-hearted solution. It was succeeded by two other RSFQ versions (ERSFQ and eSFQ, where “E/e” stands for “energy-efficient”) where *P*_S_ is totaly zero.

**Figure 5:**
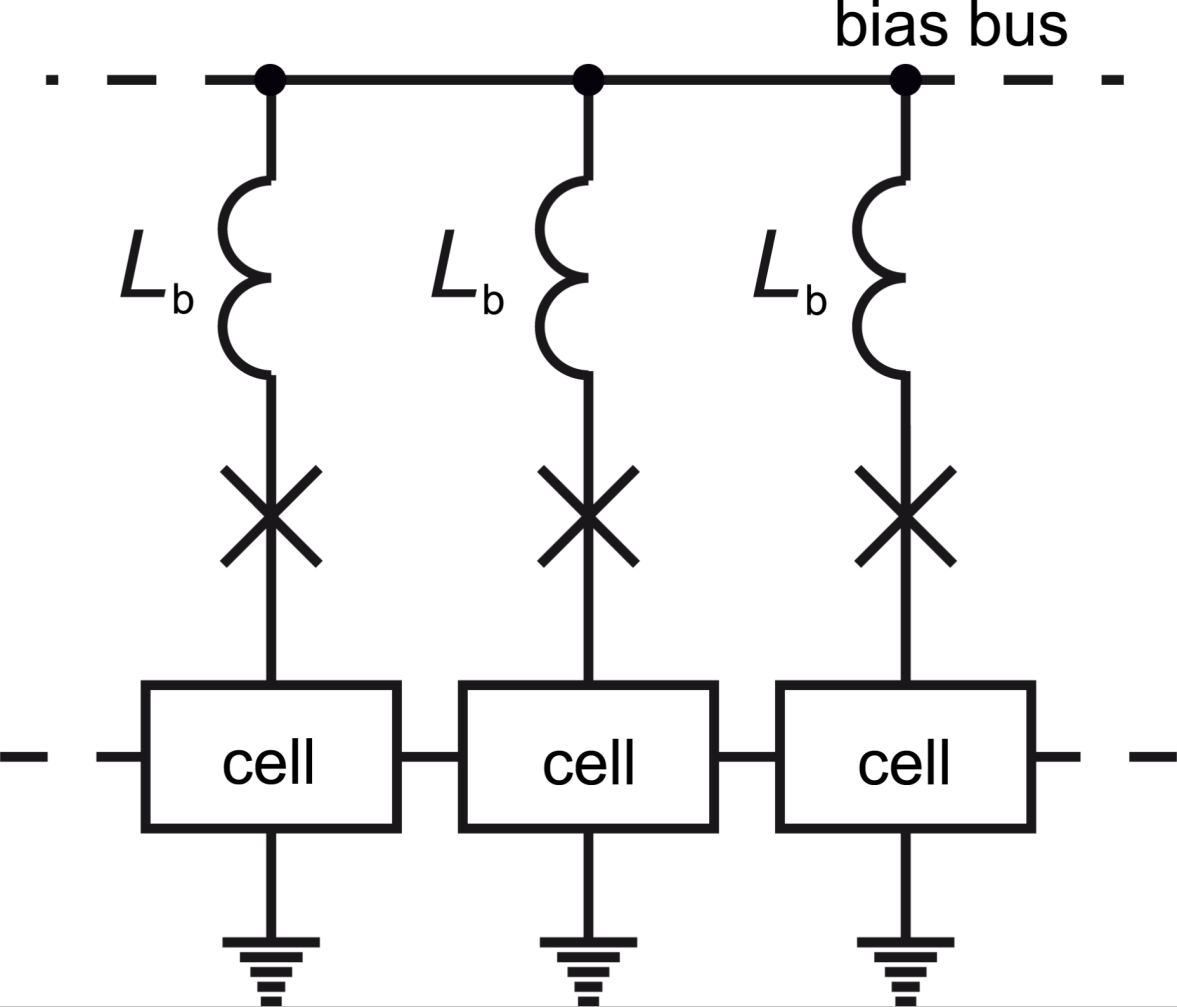
ERSFQ power supply scheme. *L*_b_ is the inductance limiting the bias current variation.

**Energy-efficient RSFQ:** ERSFQ [37] is the next logical step after LV-RSFQ. Resistors in feed lines are substituted with Josephson junctions limiting the bias current variation in this logic, see Figure 5. This replacement is somewhat analogous to the one that was done for resistors connecting SQUID cells in the very first RSFQ circuits. It provides the possibility for the circuits to be in a purely superconducting state.

The main difficulty in the elimination of bias resistors is the formation of superconducting loops between logic cells. Generally, logic cells are switched asynchronously depending on processing data. Average voltage and total Josephson phase increment are different across them. This results in the emergence of currents circulating through neighboring cells. Being added to the bias current, these currents prevent correct operation of the circuits. The imbalance of the Josephson phase increment is automatically compensated by corresponding switchings of Josephson junctions placed in ERSFQ feed lines. Since these switchings are not synchronized with the clock, some immediate alteration of the bias current is still possible. This alteration Δ*I* ≈ Φ_0_/*L*_b_ is limited by an inductance *L*_b_ connected in series with a Josephson junction in the feed line. While large value of this inductance *L*_b_ minimizes the bias current variation, its large geometric size increases the circuit area (similar to LV-RSFQ). Possible solutions of this problem are an increase of the number of wiring layers and/or the utilization of superconducting materials having high kinetic inductance. These materials can be also used for further miniaturization of logic cells themselves [19].

**Figure 6:**
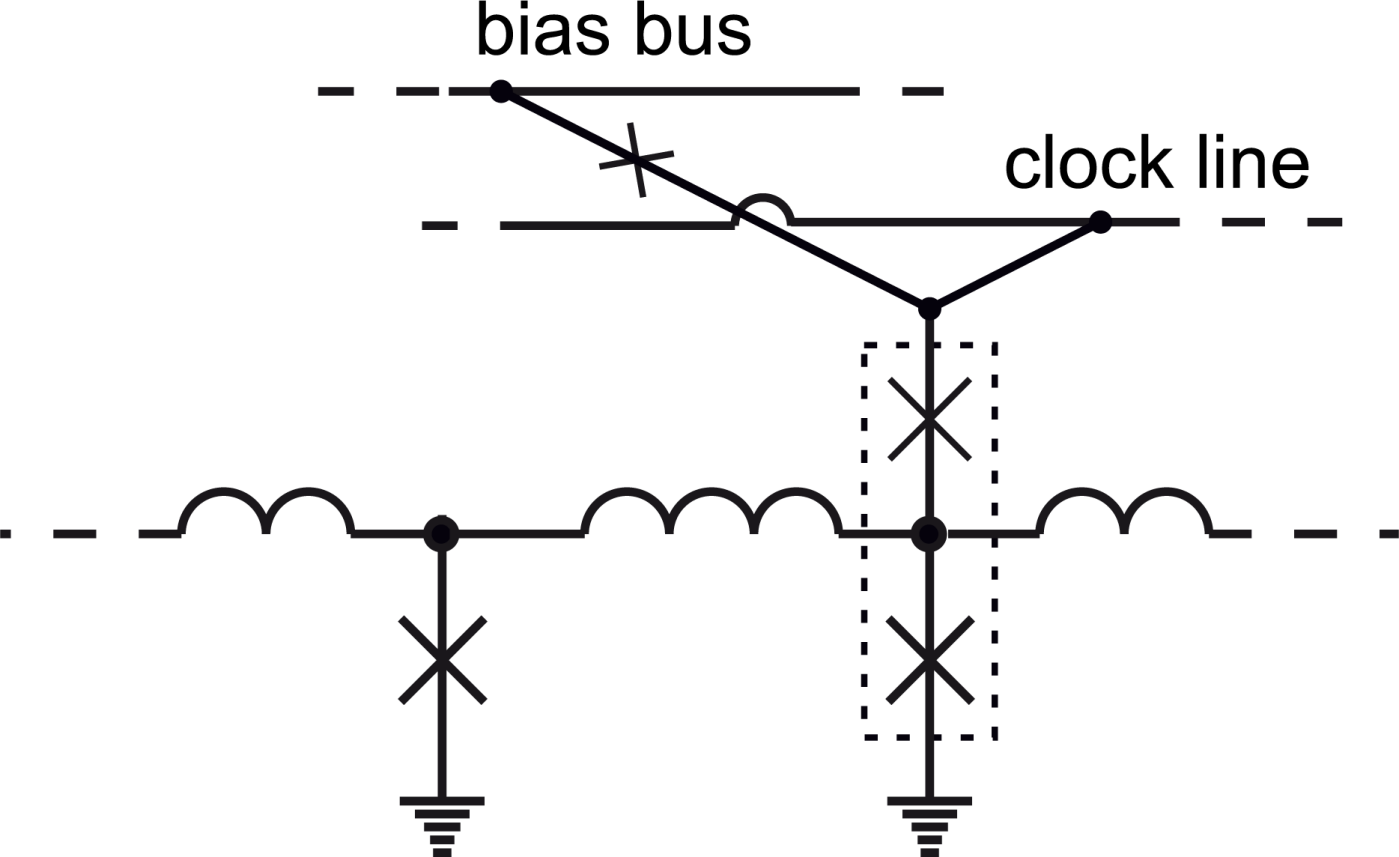
eSFQ power supply scheme. The dotted rectangle marks the decision-making pair.

**Energy-efficient SFQ:** Another energy-efficient logic in the RSFQ family is eSFQ [11,38–40]. The main idea here is the “synchronous phase balancing”. A bias current is applied to the decision-making pair, see Figure 6. One Josephson junction of this pair is always switched during a clock cycle regardless data content. Therefore, average voltage and Josephson phase increment are always equal across any such pair. This prevents the emergence of parasitic circulating currents. The Josephson junction in the feed line is required only for the adjustment of a proper phase balance during the power-up procedure. “It is not expected to switch during regular circuit operation” [11].

The achieved phase balance allows one to remove large inductances from ERSFQ feed lines, and so eSFQ circuits occupy nearly the same area as RSFQ circuits. One should note that despite of the “synchronous” nature of this logic, a method for the design of eSFQ-based asynchronous circuits was proposed in [39], making it suitable for wave-pipelined architectures.

Since the RSFQ library was designed without regarding synchronous phase balancing, the transition to eSFQ requires a correction. In some cases this leads to an increase of the number of Josephson junctions. For example, JTL should be replaced by a shift register [41] or by “Wave JTL” [39], or by one of its asynchronous counterparts: a ballistic transmission line based on unshunted Josephson junctions [42–43] or a passive microstrip line. The similarity of ERSFQ and eSFQ approaches enables an estimated total increase in the number of Josephson junctions up to 33−40% compared to RSFQ circuits [11]. The inheritance of basic cell design of RSFQ by ERSFQ makes it easier to use.

**Figure 7:**
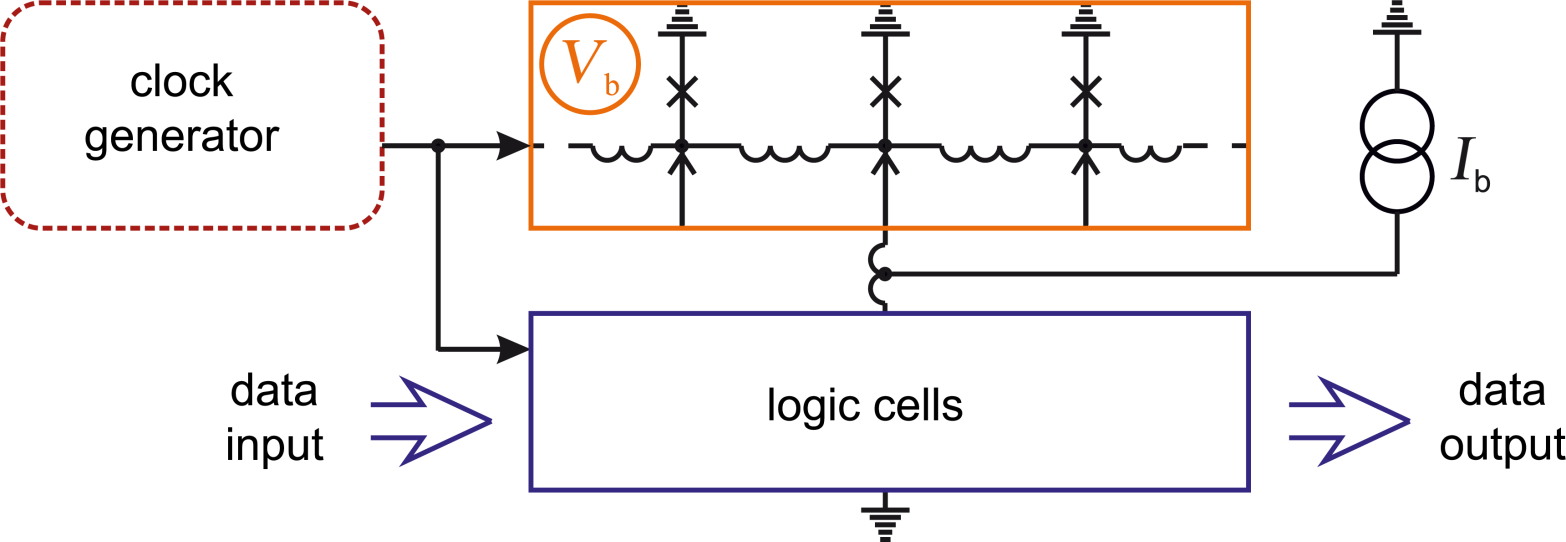
Realization of a dc bias voltage source in RSFQ circuitry.

**Common features of the RSFQ logic family:** The clock signal is effectively a part of data in ERSFQ circuits. This means that they are globally asynchronous. Since the clock frequency is determined by the repetition rate of SFQs in the clocking JTL, it can be adjusted “in flight” by logic cells according to the processed data. The bias voltage source can be implemented as a JTL fed by a constant bias current, for which the input signal is the SFQ clock applied from an on-chip SFQ clock generator, see Figure 7. The average voltage on this JTL is proportional to the clock frequency, 
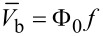
, according to the ac Josephson effect. A clock control by logic cells allows the voltage to be adjusted or even turned off. The last option corresponds to circuits switching into a “sleep mode” in which the power dissipation is zero. The realization of this power-saving mechanism at the level of individual circuits is possible through partitioning the circuits into a serial connection of islands with equal bias current but different bias voltage [44].

Since logic cells are fed in parallel in RSFQ circuitry, the total bias current increases proportional to the number of Josephson junctions. For one million Josephson junctions the bias current value could be unreasonably high *I*_b_ ≈ 100 A. The partitioning of circuits keeps it at an acceptable level [45] below 3 A.

**Figure 8:**
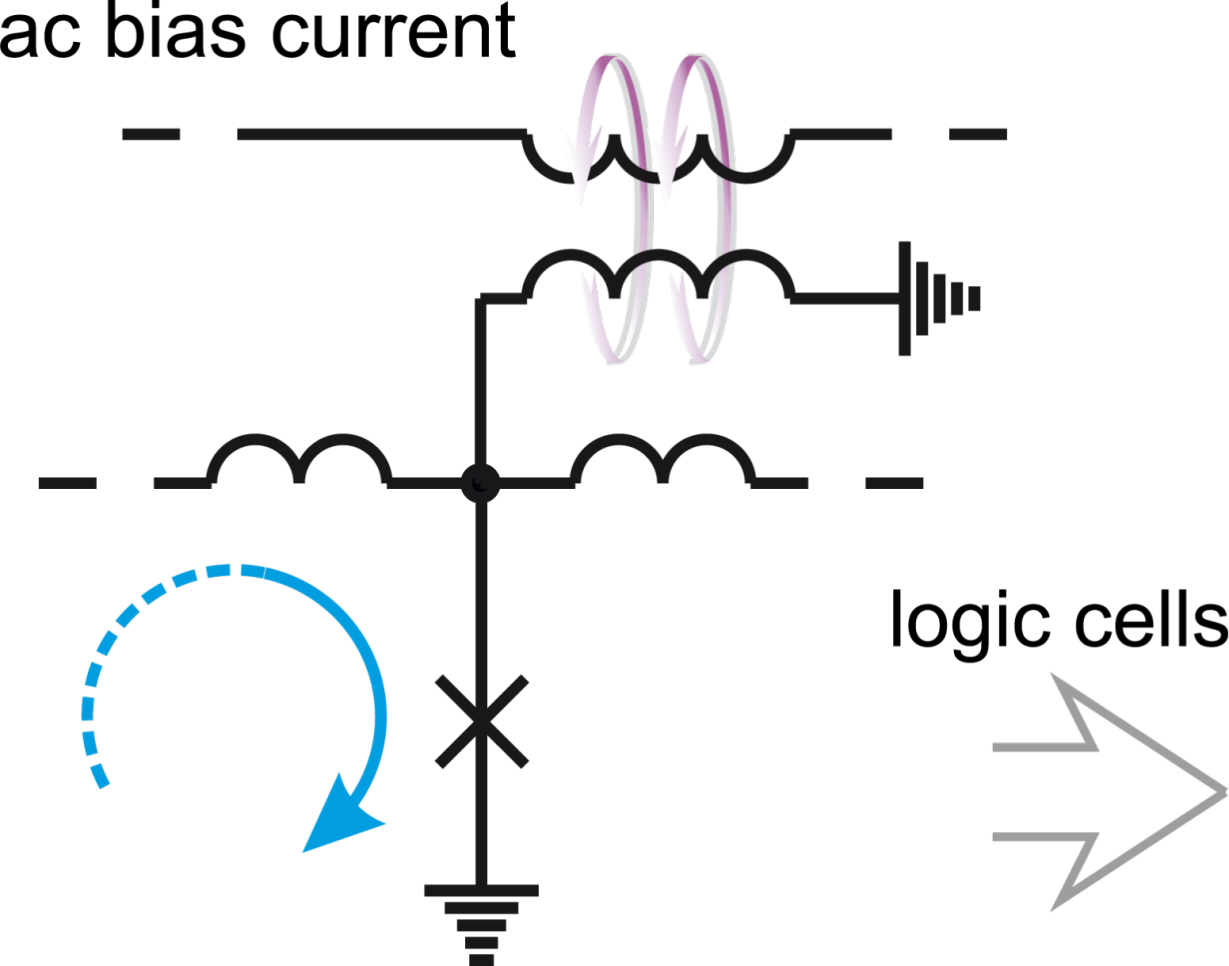
RQL ac power supply scheme. The blue arrow shows the SFQ current, violet arrows present magnetic coupling.

**Reciprocal quantum logic:** RQL was proposed in about 2008. It was developed as an alternative to conventional RSFQ, and presented as “ultra-low-power superconductor logic” [46]. The main difference between RQL and RSFQ is the power supply scheme [47]. While in RSFQ a dc power is applied to Josephson junctions in parallel through bias resistors (Figure 4), in RQL an ac power is applied in series through bias transformers, see Figure 8.

The proposed power supply scheme possesses a number of advantages: (i) No dc bias current and no bias resistors means zero static power dissipation inside the cryogenic cooler. The bias current is terminated off the chip at room temperature. (ii) A well-known design problem of RSFQ circuits is the large magnetic field of the returning bias current affecting the logic cells. It is recommended [45] to keep the maximum bias current below 100 mA in the RSFQ feed line. This return current is completely absent in RQL due to the mentioned off-chip bias current termination. (iii) Serial bias supply allows one to keep the bias current amplitude at a fairy low level [46] of the order of *I*_b_ ≈ 1.8 mA independent on the number of Josephson junctions on a chip. There is no need for large-scale circuit partitioning. (iv) The bias current plays the role of the clock signal. There is no need for an SFQ clock distribution network. (v) The clock signal is not affected by thermal noise.

**Figure 9:**
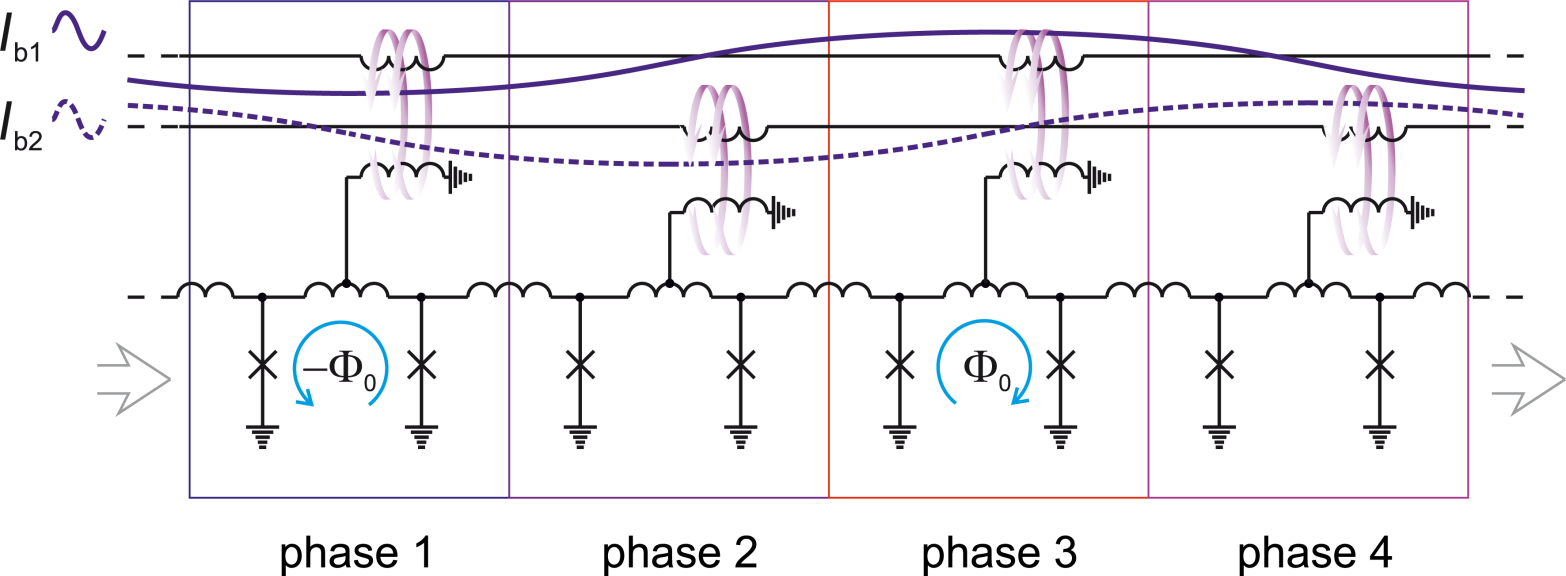
RQL transmission line with four-phase bias. *I*_b1,b2_ are ac bias currents providing the power supply and acting as clock signal. The blue arrows show SFQ currents, violet arrows present magnetic coupling.

Logical unity (or zero) is represented by a pair of SFQs having opposite magnetic flux directions (or the lack thereof) in RQL circuits. These SFQs can be transferred in one direction through the application of inversely directed bias currents, see Figure Figure 9. The SFQs are placed in the positive/negative ac current wave half period, accordingly. Unfortunately, one ac bias current is insufficient for directional propagation of the SFQs. It can provide only periodic space oscillations of the flux quanta. RQL uses two ac bias currents with a phase shift of π/2. RQL cells are coupled to these two feed lines in rotation (Figure 9). This coupling yields a space division of total bias current/clock signal into four windows shifted by 0, π/2, π, and 3π/2 wave period. By analogy with a four-stroke carburetor engine, this four-phase bias scheme provides the directionality of the SFQs propagation [46].

Logic elements connected to a single ac bias line within a single clock phase window form a pipeline. A pipeline in RQL can contain an arbitrary number of cells. One can increase the depth of the pipeline at the cost of a clock frequency decrease. The time delay of the pipeline should be less than one-third of the clock period for proper circuit operation. The circuit speed is effectively a product of clock frequency and pipeline depth. The maximum clock frequency of RQL circuits can be estimated as *f*_max_ ≈ 17 GHz under the assumption of a characteristic frequency of the Josephson junction, ω_c_/2π = 350 GHz and *N* = 8 Josephson junctions in the pipeline [47]. The RQL biasing scheme provides a self-synchronization of data. Early pulses wait at the pipeline edge for the bias current rise in the next phase window. SFQ jitter is accumulated only inside one pipeline and, therefore, timing errors are negligible, which is in contrast to RSFQ.

RQL logic cells are state machines similar to RSFQ logic cells. The internal state of a logic cell can be changed by an SFQ propagating in front of the clock wave. Its paired SFQ with opposite polarity serves for the state resetting at the end of a clock period. A complete set of RQL logic cells comprises just three gates which are AND–OR gate, A-NOT-B gate and set–reset latch. These gates behave as combinational logic cells similar to their semiconductor counterparts [47]. This makes RQL circuits design to be closer to CMOS technology than to RSFQ.

Particular RQL drawbacks as well as advantages come from the power supply scheme. A proper power supply requires high-frequency power splitters. These splitters often occupy quite a large area. For example, in the implementation of an 8-bit carry-look-ahead adder they cover an area that is ca. 2.5-times larger than the adder itself [48]. The power supply through transformers also limits the possibility for the miniaturization of the circuits. Multiphase ac bias leads to known difficulties for high-frequency design, e.g., clock skew. This practically limits the clock frequency to 10 GHz, while RSFQ circuits routinely operate at a frequency of 50 GHz. Moreover, the implementation of MCM technology becomes complicated with RQL due to a possible asynchronicity of chips or a clock phase shift. Besides inconveniences due to the high-frequency clock supply from an off-chip source, clocking by ac bias currents eliminates the possibility of clock control by logic cells. Corresponding power save mechanisms cannot be realized in RQL. In addition, one should mention RF losses in microstrip resonators, which typically make up to 50% of the total power budget even at relatively low frequencies.

The total power dissipation of RQL and ERSFQ circuits in the active mode seems similar. Static power dissipation is absent. Dynamic power dissipation is associated with the switching of Josephson junctions in data propagation processes. In RQL circuits, the Josephson junction is doubly switched for the transfer of logical unity and zero times for the transfer of a logical zero. In ERSFQ both unity and zero are transferred through switching of one of the Josephson junctions in the decision-making pair. By assuming an equal number of zeros and ones in the data, one comes to a roughly equal estimation for energy dissipation in both RQL and ERSFQ logics [19]. A more detailed analysis shows that only adiabatic switching of logic cells improves the energy efficiency of superconducting circuits markedly [19].

#### Adiabatic superconductor logic

Considered variants of superconductor logics have been proposed for non-adiabatic irreversible computation. Here, logical states are separated by an energy barrier, *E*_w_ ≈ 10^3^–10^4^*k*_B_*T* ensuring proper circuit operation. Note that the energy barrier in semiconductor circuits is two to three orders higher, *E*_w_ ≈ 10^6^*k*_B_*T*. The minimal energy barrier corresponds to Landauer’s “thermodynamic limit” [49], *E*_min_ = *k*_B_*T* ln 2. In this limit, the distinguishability of logic states is completely lost due to thermal fluctuations [12].

The energy required to perform a non-adiabatic logic operation can be estimated as the energy of transition between logical states corresponding to *E*_w_. In considered superconductor logics it is the energy of switching a Josephson junction, *E*_J_ ≈ 2 × 10^−19^ J. While presuming logical irreversibility, this energy can be lowered down to *E*_min_ ≈ 4 × 10^−23^ J (at *T* = 4.2 K) by using adiabatic switching process. Note that the Landauer limit *E*_min_ in this context reflects the entropy change in the computing system associated with an irreversible operation [49]. At the same time, there is no such limit for physically and logically reversible processes. Therefore, the energy dissipated per logical operation can approach zero in adiabatic reversible circuits.

The first ever practical reversible logic gates were realized recently [50] on the basis of adiabatic superconductor logic. The history of ASL development has begun even before the invention of RSFQ with the proposition of a “parametric quantron” [51] in 1976. This cell itself was proposed even earlier in 1954 as “rf parametron” [52], though for a different operating regime. It is interesting to note that the manner of parametric quantron cell operation was implemented later in a single-electron device [24,53] in 1996. The “single-electron parametron” operation was in fact quite similar to the ones of quantum-dot cellular automata (QCA) which were proposed for computation those years [54].

**Figure 10:**
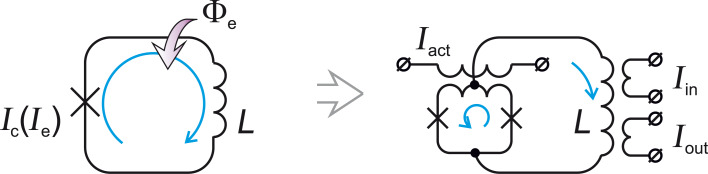
Notional (left) and practical (right) schematic of a parametric quantron. The cell state is conditioned by bias flux Φ_e_ and current *I*_e_ controlling the critical current *I*_c_ of the Josephson junction. *L* is the loop inductance. In practice, the single Josephson junction is substituted with a SQUID controlled by the activation current *I*_act_. *I*_in_/*I*_out_ are input/output currents.

**Basic principles of operation of an ASL circuit:** A parametric quantron is a superconducting loop with a single Josephson junction shown on the left-hand side of Figure 10. Its state is conditioned by external magnetic flux, Φ_e_, and current, *I*_e_, controlling the critical current of the Josephson junction *I*_c_(*I*_e_). The potential energy of this cell is a sum of the Josephson junction energy, *U*_J_ = (*E*_J_/2π)[1 − cos φ] (followed directly from the dc Josephson effect), and the magnetic energy, *U*_M_ = (*E*_J_/2π)[φ − φ_e_]^2^/2*l*:

[2]
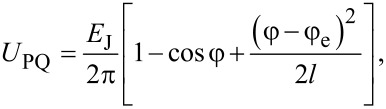


where φ_e_ = 2πΦ_e_/Φ_0_, *l* = 2π *I*_c_*L*/Φ_0_ is the normalized loop inductance.

**Figure 11:**
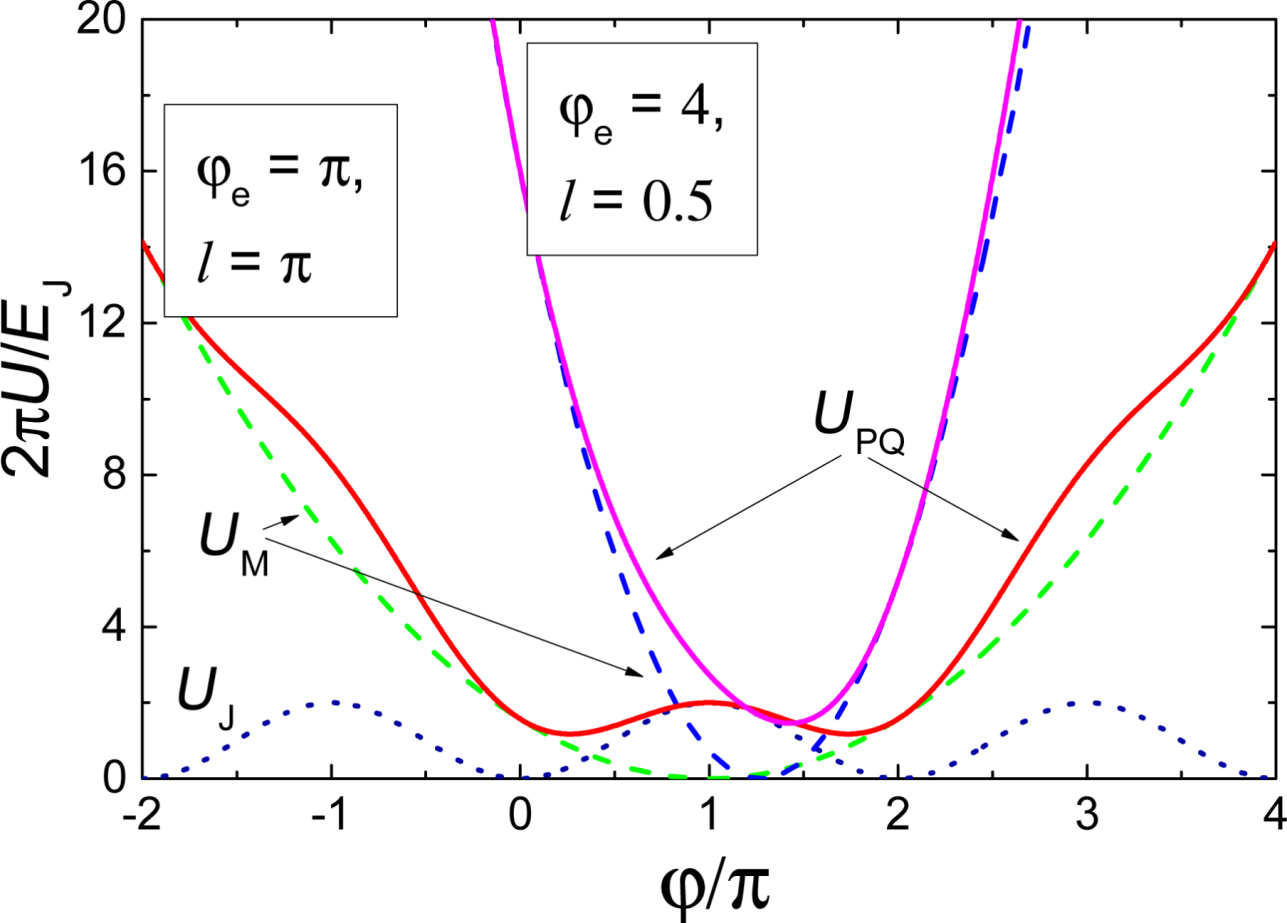
Potential energy of a parametric quantron *U*_PQ_ (Equation 2) (solid lines) and its terms: magnetic energy *U*_M_ (dashed lines), and Josephson junction energy *U*_J_ (dotted line).

It is seen that the external parameters Φ_e_ and *I*_e_ control the vertex (through φ_e_[Φ_e_]) and slope (through *l*[*I*_c_(*I*_e_)]) of the parabolic term of the potential energy *U*_M_ in Equation 2. Under an appropriate bias flux, φ_e_ ≈ π, the potential energy of the parametric quantron *U*_PQ_(φ) can take a single-well (for *l <* 1) or a double-well (for *l >* 1) shape depending on *I*_e_, see Figure 11. Logical zero and unity can be represented by the cell states with the phase of the Josephson junction, φ, lower or higher than π. For *l >* 1 these states correspond to minima of the potential wells. Physically they correspond to a different magnetic flux in the loop (with currents circulating in the loop in the opposite directions if φ ≠ 2π*n*, where *n* is an integer).

**Figure 12:**
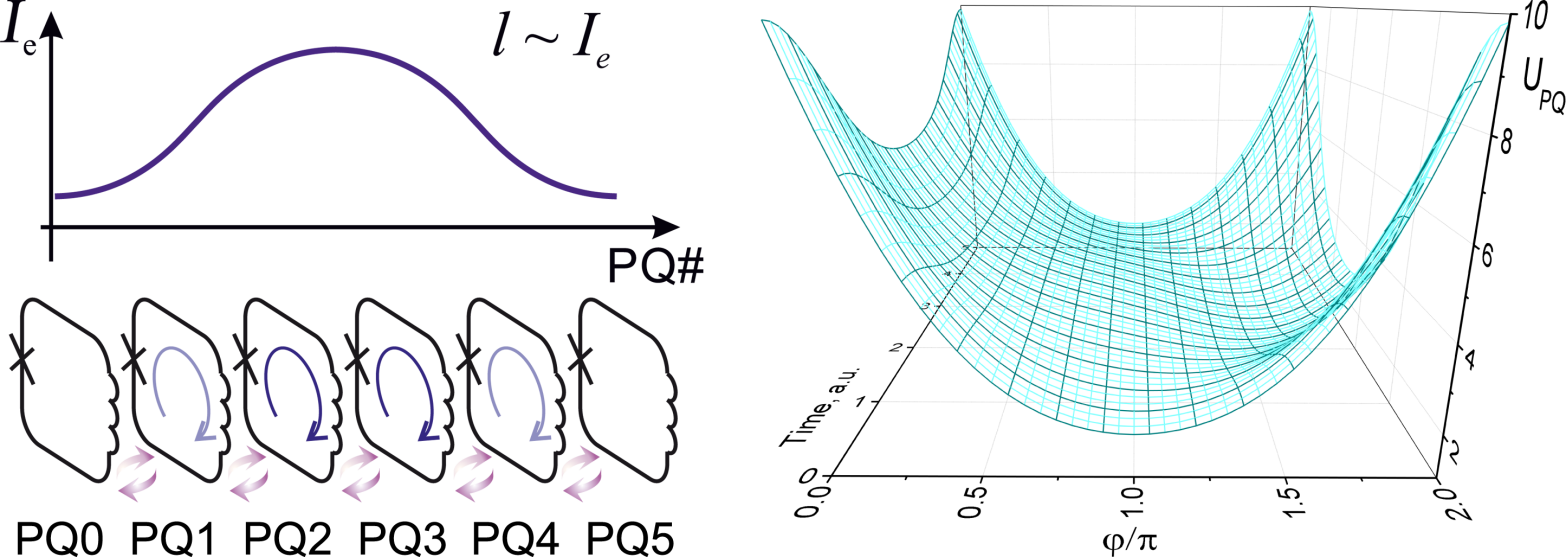
Logical state transfer in an array of magnetically coupled parametric quantrons under a driving current pulse *I*_e_, with corresponding change of the potential profile of a single cell in time. Violet arrows represent magnetic coupling.

A logical state transfer can be performed in an array of magnetically coupled parametric quantrons biased into the working point φ_e_ = π. Current pulses *I*_e_ should be applied sequentially to the cells increasing their normalized inductance one by one, see Figure 12. A logical state can be shared by a group of cells, wherein it is most pronounced in a cell with the largest *l* at a particular point in time. Dynamics of this transfer process can be made adiabatic by adjusting the shape of the driving current pulse *I*_e_. Cross-coupling of the cells enables adiabatic reversible logic operations [55].

The single Josephson junction of parametric quantron is substituted by a SQUID (see right-hand side of Figure 10) in practical implementations [56]. Here, the activation current, *I*_act_, plays a role similar to the one of *I*_e_. It induces a circulating current in the activation SQUID, and therefore increases the phases of the Josephson junctions according to the dc Josephson effect. This in turn corresponds to an increase in the energy in the Josephson junctions, which can be minimized with the appearance of a current circulating in the main parametric quantron loop (the loop containing the inductance *L*). However, the states with both directions of the circulating current are equally favorable due to symmetry of the scheme. The choice of one of these states is made through the direction of the input current *I*_in_ playing the role of Φ_e_ here. Due to the fact that a current-less state is unstable, corresponding to a local maximum of the potential energy, the current *I*_in_ can be infinitesimally small. A parametric quantron can provide a virtually infinite amplification of the magnetic flux, accordingly. Since a potential energy minimum is achieved with circulating currents in both: activation SQUID and main parametric quantron loop, it was noted that the roles of the activation and the input/output can be swapped [57].

Unfortunately, already the first designs of a processor based on physically and logically reversible parametric quantron in the mid-1980s [56] showed this approach to be impractical. The reason was as follows: logical reversibility can be achieved by temporary storage of all intermediate results [58]. Together with the predominance of short-range interactions this produces severe hardware overhead. Indeed, the realization of an 8-bit 1024-points fast convolver required almost 10^7^ parametric quantrons [56]. About 90% of them were operated just as elements of shift registers, transferring data through the processor [56]. It was noted that such circuits are also characterized by low speed (in comparison to RSFQ) and low tolerance to parameter variations [24].

**Circuits based on quantum flux parametrons:** A few years after the works on reversible circuits, the same principles of operation were utilized for the development of a generally non-reversible Josephson supercomputer. In this effort the parametric quantron was renamed as “quantum flux parametron” (QFP) [57]. The major problem of QFP-based circuits was high-frequency multi-phase ac power supply (which was later borrowed by RQL). While there were different approaches elaborated for its solution [57,59], finally multi-phase ac biasing was recognized to be an intractable obstacle for the implementation of complex practical high-clock-frequency circuits and the QFP-based approach was abandoned for some years.

Renewed interest to ASL was introduced by the development of superconductor quantum computers. QFPs are utilized as qubits and couplers in adiabatic quantum optimization systems of D-Wave Systems [60–61]. Another reason for the current rise of interest to ASL is the Japanese JST-ALCA project “Superconductor electronics system combined with optics and spintronics” [62]. The idea of the project is the development of an energy-efficient supercomputer based on the synergy of the mentioned technologies. The superconductor processor of the computing system is planned to be based on QFPs operated in the adiabatic regime. The processor prototype has an 8-bit simplified RISC architecture and features ca. 25000 Josephson junctions and about 10 instructions. In this context, adiabatic operation of QFP was investigated in order to reduce the dynamic energy consumption down to the fundamental limit [63]. Adiabatic QFP was abbreviated as AQFP in these works [50,63–68].

AQFP-based circuits were tested experimentally [64] at a clock frequency of 5 GHz showing energy dissipation at the level of 10^−20^ J. Theoretical analysis reveals that AQFP can be operated with energy dissipation below the thermodynamic limit [65]. The product of energy dissipated per clock cycle on a cycle time could approach the quantum limit [68] at 4.2 K cooling temperature, with utilization of standard manufacturing processes [69]. Comparison of AQFP-based design with designs based on CMOS FPGA, for example for the implementation of the Collatz algorithm, showed that the former is about seven orders of magnitude superior to its counterpart in energy efficiency, even including the power of cryogenic cooling [13]. AQFP-based logic cells can be implemented by combining only four building blocks: buffer, NOT, constant, and branch [67]. Together with the AQFP latch [66] these blocks enable the design of an adiabatic circuit of arbitrary complexity. Recently, a 10000 gate-scale AQFP circuit was reported [70].

Magnetic coupling of AQFP gates is performed via transformers. The current flowing through the transformer wire must not be too small because it ought to provide appropriate bias flux to subsequent cell despite of a possible technological spread of AQFP parameters. This limits the maximum wire length to about 1 mm [67]. This length is further conditioned by a trade-off with maximum clock frequency, which is limited to 5 GHz in practical circuits [50,63–68]. This clock frequency limitation relaxes the complexity of the design of ac bias lines. However, with circuit scale increase, the lengthy distribution of clock lines is nonetheless expected to generate a clock skew between logic cells [13].

While adiabatic circuits are clearly the most energy-efficient, their operation frequency is relatively low and the latency is relatively large. However, recently it was shown that due to the intrinsic periodicity of the potential energy of AQFP, the cell can be operated at double or even quadruple activation current frequencies with an increase of the current amplitude [71]. This opens the opportunity to speed up AQFP circuits up to clock frequencies of 10 GHz or even 20 GHz.

**nSQUID-based circuits:** Above, we already mentioned that it is possible to swap the roles of activation current and input/output in the parametric quantron. In this case, information is represented by the magnetic flux of the SQUID, while its bias current flowing through the main parametric quantron loop plays the role of excitation. It was noted that while the SQUIDs of such cells may be coupled magnetically, their activation current pulse can be provided sequentially using a common bias bus. For better control of the SQUID state in this scheme the value of the main parametric quantron loop inductance should be minimized. In addition, it was proposed to provide negative mutual inductance between the two parts of the SQUID loop inductance [72]. A SQUID with negative mutual inductance is called “nSQUID”. Its inductance is effectively decreased for the bias current but increased for the current circulating in its loop.

**Figure 13:**
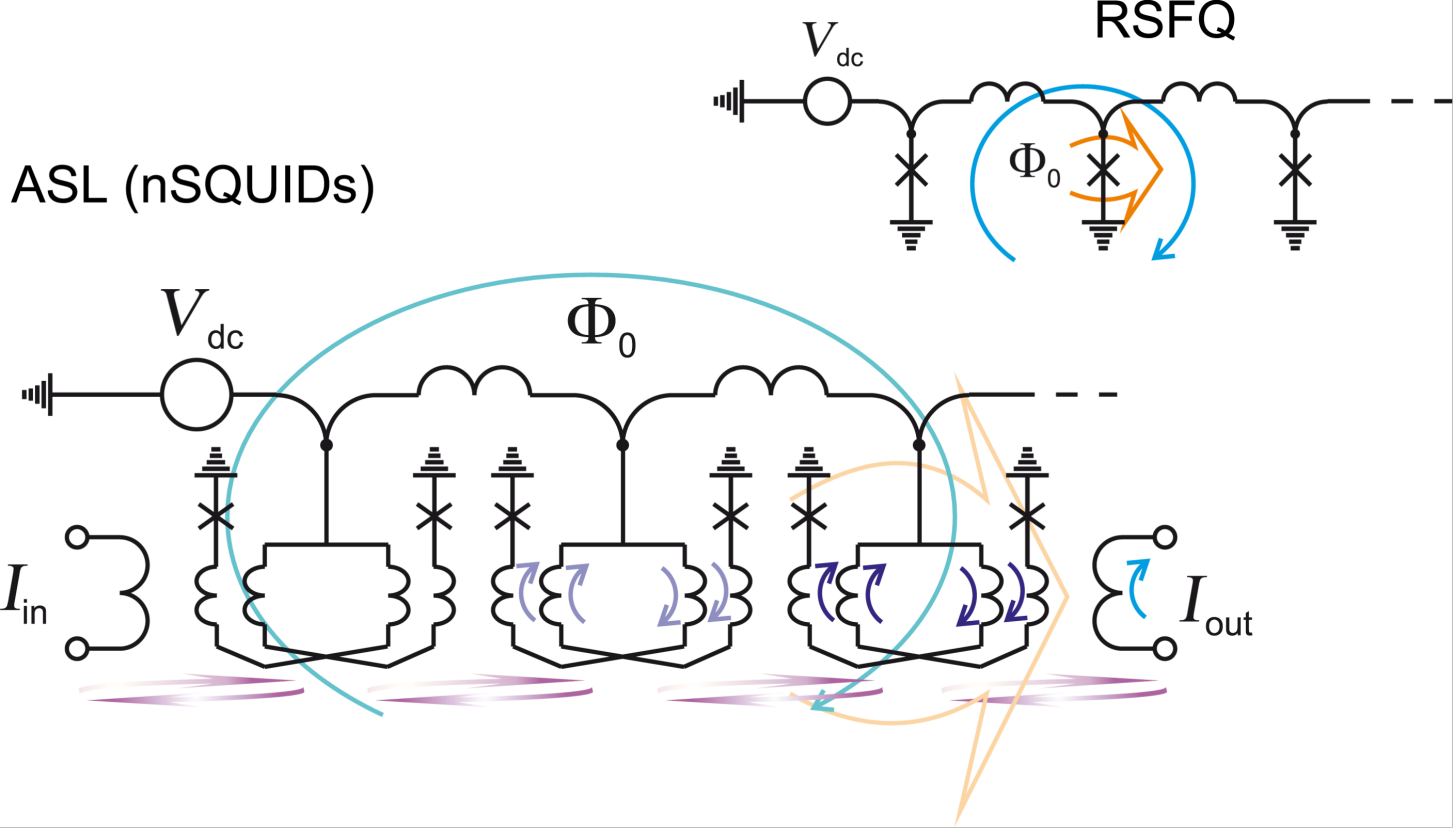
nSQUID-based adiabatic data bus and RSFQ data bus. Blue arrows show circulating currents, orange arrows highlight critically biased elements, violet arrows present magnetic coupling.

The successive application of activation current pulses to nSQUIDs from a common bias bus can be realized by using an SFQ [72–75]. Note that an nSQUID-based transmission line is quite similar to conventional RSFQ JTL with the substitution of Josephson junctions by nSQUIDs, see Figure 13. Here, a data bit is spatially bound to SFQ. This way of application of activation current pulsea allowed for the switch from ac to dc power supply. It was shown that it is possible to switch also from magnetic to galvanic coupling between nSQUIDs [73].

nSQUID-based circuits were successfully tested [74] at a clock frequency of 5 GHz. At a lower frequency, 50 MHz, their energy dissipation per logic operation was estimated [11] to be close to the thermodynamic limit, ca. 2*k*_B_*T* ln 2. Since nSQUID circuits utilize SFQ clocking, the clock rate (and hence, the power dissipation) can be adjusted “in flight” like in RSFQ circuits. Note that the energy associated with SFQ creation or annihilation *E*_J_ is much greater than the thermodynamic limit at 4.2 K. SFQs are “recycled” to avoid this energy dissipation. For this purpose the circuits are made in a closed-loop manner as “timing belts” [75]. Thus, total number of SFQs remains unchanged. However, this imposes certain restriction on the circuit design. It is interesting to note that it was proposed to use nSQUID circuits for the implementation of “flying qubits” transmitting quantum information [73,76]. Yet, this idea is not implemented experimentally.

#### Discussion

We considered non-adiabatic and adiabatic logics the implementations of which are different mainly in the type of power supply: ac or dc. Each type has its own advantages and disadvantages. The most attractive feature of ac versus dc bias is that the power is supplied in series. We should note that this feature can be utilized also for dc-biased circuits by using ac-to-dc converters [77]. At a particular frequency of the ac power source the required bias voltage can be obtained by serial connection of these converters. The power supply of different parts of a large-scale dc-biased circuit by such voltage sources could eliminate the need for circuit partitioning. In general, SFQ ac-biased circuits are good for the design of large regular structures. The largest superconductor digital circuit is an ac-biased shift register containing 809000 Josephson junctions [78]. It was used as a fabrication process benchmark circuit like a kind of “scan chain”.

A dc power source is most convenient in terms of providing the power into the cryogenic system. Indeed, the bandwidth of microwave cables is often narrow to prevent heat inflow. In order to overcome the limitation on the maximum frequency of ac-biased circuits it was proposed to use a dc-to-ac converters as on-chip power source [79]. This converter was successfully tested in experiment providing an oscillation frequency of 4.4 GHz. The output ac bias current amplitude can be tuned by varying the dc bias current of the convertor. Utilization of ac-to-dc and dc-to-ac converters allows for the use circuits based on different logics on a single chip, increasing the variability of the design.

Physical localization of information corresponding to quantization of magnetic flux leads to another issue, especially in digital SFQ circuits. Due to low gain from a Josephson junction, the circuits are featured by low fan-out. An SFQ has to propagate through a large and slow SFQ splitter tree to split information into multiple branches. The same situation is with merging of multiple outputs. A solution of this problem can be found in the utilization of magnetic control over cells by using a current control line. This approach can be realized with SFQ-to-current loop converters [80–81]. A similar technique can be used in merging of multiple outputs [82].

SFQ-to-current conversion can be realized also by superconducting–ferromagnetic transistors (SFTs) [83] or by “non-Josephson” devices such as an n-Tron [84]. The former is a three (or four) -terminal device comprising two stacked Josephson junctions. One of them, the “injector”, (containing ferromagnetic layer(s)) serves for injection of spin-polarized electrons in a common superconducting electrode of both junctions, thus suppressing its superconductivity. This manifests itself as redistribution of the superconducting current flowing through this electrode or as degradation of the critical current of the “acceptor” (typically SIS junction) depending on the configuration of the device [85]. While having good input/output isolation, SFT is capable of providing voltage, current and power amplification.

An n-Tron is a three-terminal device comprising a superconducting strip with a contraction in the middle to which the third terminal tip is connected. A current pulse from the third terminal switches off the superconductivity of the nanowire, that is similar to SFT operation to some extent. Unlike a Josephson junction, the nanowire in the resistive state possesses several kiloohms of resistance, which provide a high output impedance and a high voltage signal [86–87]. Both devices can be utilized as an interface [85,88–89] between superconductor circuit and CMOS electronics or memory depending on requirements to output signal and energy efficiency.

It is well known that the major parts of computation time and power consumption are associated with communications between logic and memory circuits [90]. Logic cells possessing internal memory are now being considered as a possible element for the development of new, more efficient computers [91–92]. Superconductor logic circuits utilizing their internal memory were named “MAGIC” (Memory And loGIC) circuits [90,93]. This concept is based on conventional ERSFQ cells involving their renaming or rewiring. It promises an increase in clock rate to above 100 GHz, combined with an up to ten-fold gain in functional density. In general, the mentioned localization of information and high non-linearity of Josephson junctions make superconductor circuits to be ideally suited for the implementation of unconventional computational paradigms like cellular automata [94–95], artificial neural networks [96–98] or quantum computing [99–103].

Unfortunately, the major problem of superconductor circuits does not relate to a particular logic of computation. Low integration density in all cases limits complexity, and therefore performance of modern digital superconductor device. Possible solutions here are miniaturization of existing elements and increase of their functionality. The former can be obtained by scaling down the SIS Josephson junction [104], or search for other high-accuracy technological processes providing nanosized junctions with high critical current density and normal-state resistance [104–106]. Another direction of the research is the substitution of the conventional loop inductance with a kinetic inductance or the inductance of the Josephson junction [19]. This also allows one to make the circuits more energy efficient. Indeed, the critical current *I*_c_ and the loop inductance *L* of a Josephson junction are linked in SFQ circuits. Their product should be *I*_c_*L* ≈ Φ_0_ for proper operation. The critical current *I*_c_ has to be decreased in order to improve the energy efficiency, *E*_J_ ≈ *I*_c_Φ_0_. This leads to an increase in the inductance making the circuit to be sparse. A miniaturization of the inductance weakens this problem. Unfortunately, the transformer remains an inherent component of the circuits which can not be miniaturized in this way. One should note that contrary to CMOS technology where the transistor layer is implemented on a substrate, Josephson junctions can be fabricated at any layer. This provides the opportunity for the utilization of 3D architectures. With the anticipated technological advances, the Josephson junction density up to 10^8^/cm^2^ seems achievable.

Finally, Josephson junctions with unconventional current-phase relation (CPR) can be utilized in a circuit for its miniaturization. For example, the so-called “π”-junction (a junction with a constant π shift of its CPR) can be used as a “phase battery” providing constant phase shift [107–108] instead of a conventional transformer. Control of the junction CPR phase shift [109] can provide the change in the logic cell functioning, e.g., converting AND to OR. This mechanism can be also used for the implementation of memory cells [109–110]. Historically, the problem of element miniaturization was first recognized in development of superconductor random access memory (RAM). Since that time, the need for dense cryogenic RAM is the major stimulus for innovative research in this area.

### Memory

Among the many attempts to create a cryogenic memory compatible with energy-efficient superconducting electronics, we want to single out the four most productive competing directions: SQUID-based memory, hybrid Josephson–CMOS memory, JMRAM and OST-MRAM.

#### SQUID-based memory

The presence or absence of SFQ(s) in a superconducting loop can be the physical basis for a digital memory element. Due to high characteristic frequency of Josephson junctions, SQUID-based memory cells stand out with fast (few picoseconds) [111] write/read times favorable for RAM, which is indispensable for data processor. Throughout various SQUID-based RAM realizations, the memory element was provided with destructive [112–114] or non-destructive [115–117] readout. Memory cells contained, accordingly, two [112] to ten [111] Josephson junctions. With micrometer-scale dimensions of the Josephson junction in the late 1990s this resulted in a memory cell area of the order of a few hundreds of micrometers squared. While the power dissipation per write/read operation was at the level of microwatts, the memory chip capacity [117] was only up to 4 kb. In this particular 4 kb RAM memory [117] the memory drivers and sensing circuits required ac power, which limited the clock frequency to 620 MHz. Later, an all-dc-powered high-speed SFQ RAM based on pipeline structure for memory cell arrays was proposed [118]. Estimation showed that this approach allows up to 1 Mb memory on a 2 × 2 cm^2^ chip operated at 10 GHz clock frequency and featuring 12 mW power dissipation. Still, it was never realized in experiment.

#### Hybrid Josephson–CMOS memory

The low integration density of SQUID-based memory cells seemed to be a significant obstacle to the development of low-temperature RAM with reasonable capacity. This approach was succeeded by hybrid Josephson–CMOS RAM in which Josephson interface circuits were amended by CMOS memory chips [119–123]. This combination allowed to develop 64 kb, 4 K temperature RAM with 400 ps read time, and 21/12 mW power dissipation for write/read operations, respectively [123]. The CMOS memory cell was composed of eight transistors. While being fabricated in a 65-nm CMOS process, the cell size was about three orders of magnitude smaller than the one of its SQUID-based counterparts. The main challenge in design of this memory system was the amplification of the sub-millivolt superconductor logic signal up to the level of ca. 1 V required by CMOS circuits. This task was accomplished in two stages. First, the signal was amplified to 60 mV using a Suzuki stack, which can be thought as a SQUID with each Josephson junction substituted by a series array of junctions for high total resistance [124]. In the second step, the reached 60 mV signal drives a highly sensitive CMOS comparator to produce the required output level.

Suzuki stack [122] and CMOS comparator [125] were optimized for best compromise of power and time performance. Their simulated power-times-delay products for read operation were 2.3 mW × 47 ps (0.11 pJ) and 6.4 mW × 167 ps (1.1 pJ), respectively. This made up to 73% and 53% of total memory system read power and time delay, correspondingly. These results lead to a severe restriction of the overall system performance by the interface circuits. Recently, it was shown that the power consumption can be significantly decreased through the utilization of energy-efficient ERSFQ decoders and n-Trons as high voltage drivers [89]. This could provide an energy efficiency improvement up to three times for 64 kb, and up to 12 times for 16 Mb memory. In the latter case, the access time of a read operation is estimated to be 0.78 ns.

While the hybrid memory approach showed better memory capacity, its power consumption and time characteristics are still prohibitive. It was summarized that for implementation of practical low-temperature RAM one should meet the following criteria [126]. (i) Scale: memory element dimensions below 100 nm (less than 200 nm pitch); (ii) write operation: 10^−18^ J energy with 50–100 ps time delay per cell; (iii) read operation: 10^−19^ J energy with ca. 5 ps time delay per cell. An idea to meet the requirements nowadays is to introduce spintronics (including superconductor spintronics) in RAM design.

#### JMRAM

It is possible to drastically reduce the size of a superconducting memory cell by using a controllable Josephson junction with magnetic interlayers instead of a SQUID [127–132]. The topology of such a magnetic Josephson junction (MJJ) is usually of two types: (i) sandwich topology which is well suited for CMOS-compatible fabrication technology, and (ii) a topology with some heterogeneity of the weak-link area of the junction in the plane of the layers. Below we present MJJ valves according to this classification.

**Figure 14:**
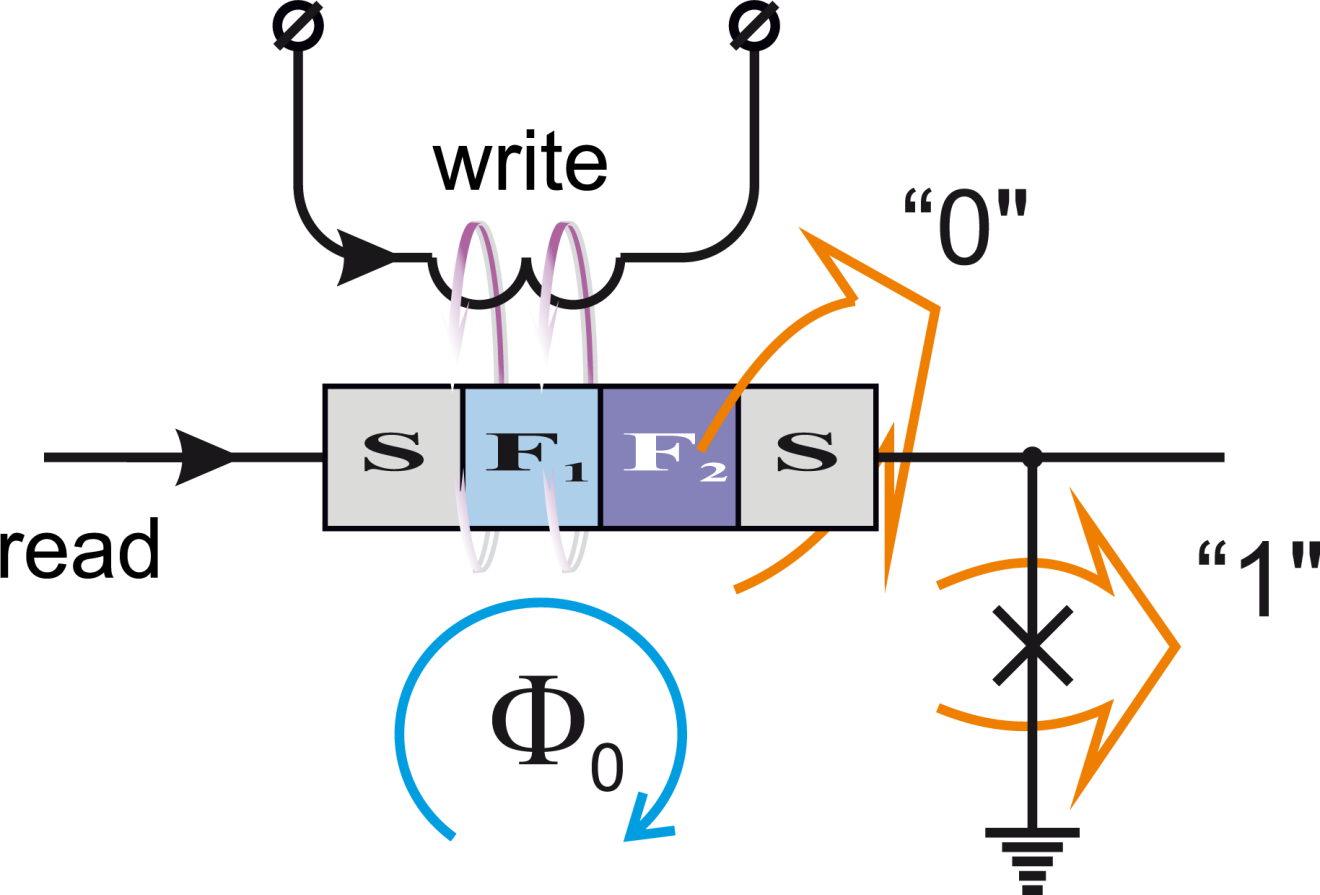
Principal scheme of implementation of write and read operations in a circuit based on MJJ valve.

**MJJ valve of sandwich topology:** The search for an optimal way of implementing compact MJJ valves is still ongoing. The most obvious solution is to use two ferromagnetic layers with different magnetic rigidity in the area of the weak link of the junction [133–135]. The critical current of such a junction is determined by effects resulting from the coexistence and competition of two orderings for electron spins, namely “superconducting” (S) (with usually antiparallel spins of electrons in the so-called “Cooper pairs”) and “ferromagnetic” (F) (with parallel ordering of electron spins). Magnetization reversal of a “weak” F-layer leads to switching between collinear and anti-collinear orientations of the F-layer magnetic moments in the bilayer. This, in turn, provides alteration in the total effective exchange energy, *E*_ex_, and hence, an effective suppression of the critical current in the MJJ. While magnetization reversal can be executed by application of an external magnetic field [136], the critical current can be read out, e.g., with the inclusion of the MJJ into a decision-making pair [137], see Figure 14. It is possible to trace some analogy between this effect and the phenomenon of giant magnetoresistance [138], which is actively used in conventional magnetic memory cells.

A common drawback of most MJJs is the small value of their characteristic frequency (ω_c_ ~ *I*_c_*R*_n_) in comparison with SIS junctions. Indeed, here one has to perform the magnetization reversal of the weak F-layer with relatively small exchange energy in order to manipulate the total critical current against the background of its considerable suppression by the strong ferromagnet. Low ω_c_ outflows in slow read operation and complicates the integration of MJJs in SFQ logic circuits. There are several approaches to solve this problem. One of them is the use of noncollinearly magnetized ferromagnetic layers [139–142]. In this case, the triplet superconducting correlations of electrons are formed in the weak-link area of the junction. A part of them are characterized by collinear orientation of electron spins in Cooper pairs. They are unaffected by exchange field of the ferromagnets, thus increasing the MJJ critical current while maintaining its normal state resistance. The “triplet” current can be controlled by external magnetic fields through magnetization reversal [143]. Still, this approach implies the implementation of a number of additional layers (and interfaces) in the structure, which reduces its critical current.

One should also note that an alteration of *E*_ex_ could result in a π-shift of MJJ CPR. In this case the valve can be utilized as controllable phase battery [109]. The inclusion of an MJJ into a SQUID loop allows for a fast read-out of its state [110]. However, here miniaturization reduces only to the replacement of the SQUID inductance with the MJJ.

Another approach is based on the localization of the magnetic field source outside the weak-link area of the Josephson junction but in the closest proximity [144–147]. For example, the F-bilayer can be placed on top of the SIS junction. In this case, a stray magnetic field penetrating into the junction area controls its critical current. If the junction S-layer neighboring the F-bilayer is thin enough, the coupling of the vector potential of the stray magnetic field to the superconducting order parameter phase could also noticeably affect the Josephson phase difference across the SIS junction. The SIS junction is utilized here just for reading out the ferromagnet state, and therefore, its characteristic frequency remains high. Still, since the strength of the magnetic field is proportional to the volume of the ferromagnet, a possibility of miniaturization of such memory element is doubtful.

A modulation of the critical current can be obtained even in a structure with a single magnetic layer by changing the value of its residual magnetization [148]. It is possible to improve the characteristic frequency by the inclusion of dielectric (I) and thin superconducting (s) layers in the weak-link area of the MJJ to increase *R*_n_ and *I*_c_, correspondingly [149–156]. Such SIsFS valves possess characteristics close to a SIS junction [151]. However, compatibility with superconductors requires the utilization of ferromagnets with relatively low coercive field, which are typically characterized by a non-square shape of the hysteresis loop. This in turn leads to an uncertainty of the MJJ critical current at zero applied magnetic field after multiple magnetization reversals. In addition, miniaturization here faces the same difficulties as in the previous approach. For these reasons, it seems especially fruitful to replace the I and F layers with two magnetic insulator IF-layers to construct a Josephson S(IF)s(IF)S valve [157–159]. Its operation relies on variable suppression of the superconductivity of the middle s-layer. Yet, this promising structure is complicated to fabricate.

**Figure 15:**
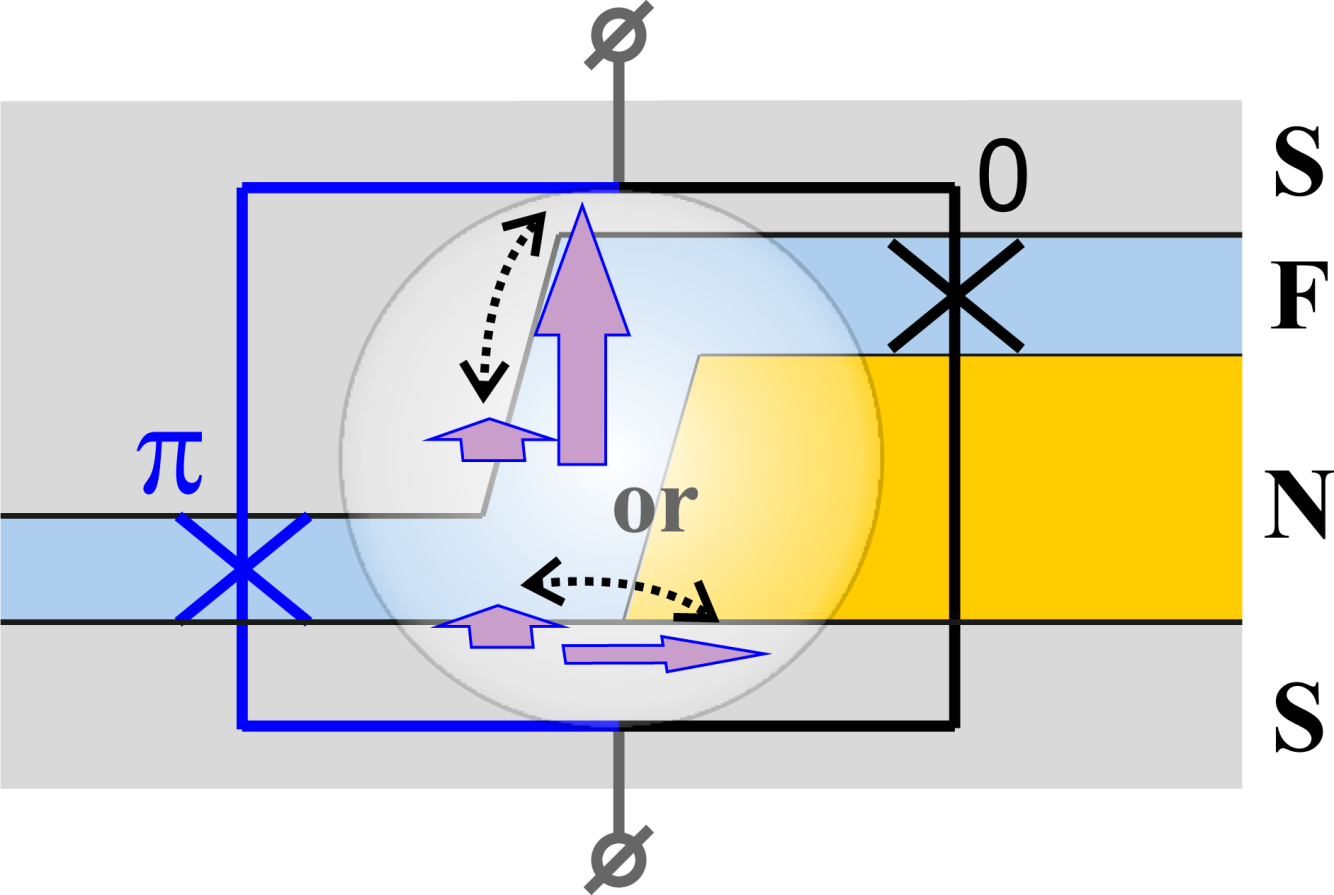
Cross section of an SF-NFS MJJ with CPRs of its parts shifted in phase by π. Arrows show F-layer magnetization directions corresponding to the on/off states of the valve.

**MJJ valve with in-plane heterogeneity of the weak-link area:** The second type of valves implies a heterogeneity of the weak-link region in the junction plane providing a separation of the structure into two parts. The CPR of these parts can be different, e.g., the conventional CPR and the one shifted in phase by π[154,160]. Such MJJ may be thought as nanoSQUID with conventional and “π”-lumped junctions. Its implementation may comprise a ferromagnetic interlayer and a sandwich containing the same F-layer and a normal metal (N) layer, see Figure 15. If the F-layer magnetization is aligned perpendicular to the nanoSQUID plane, it compensates the Josephson phase gradient across the MJJ making its critical current to be high. When the magnetization is being rotated by 90°, this effect is turned off and *I*_c_ becomes low. Here for proper operation of this SF-NFS-based MJJ the flux of residual magnetization must be comparable with the flux quantum Φ_0_.

The second common problem of MJJ-based memory is the long time for write operations. Bit write is commonly performed by magnetization reversal of at least one of the F-layers. For this reason, the recording time is of the order of the inverse frequency of ferromagnetic resonance. It is usually more than two orders of magnitude larger than the characteristic time of SIS junction switching. Thus, elimination of magnetization reversal from the operation of the valves is desired. It is worth noting that a nano-sized trap for a single Abrikosov vortex in the vicinity of Josephson junction [161–162] allows for sufficiently fast write operations. However, the energy dissipation associated with the annihilation of such a vortex (ca. 10^−18^ J) may contradict to the paradigm of energy efficiency.

This challenge can be met with MJJ having a bistable Josephson potential energy. Josephson phases of its ground states could be equal to ±φ (0 *<* φ *<* π). One can realize both write and read operations with such φ-junctions on a picosecond-timescale [163–169]. The disadvantage of this approach is the difficulties with the implementation of the φ-state. In practice, it is possible only in structures with heterogeneous weak-link regions of rather large size.

One more operation principle of MJJ valves relies on the control of the formation of superconducting phase domains [156]. The effect can be realized in SIsFS MJJ with the sFS part substituted with, e.g., a heterogeneous SF-NFS combination. The middle s-layer is broken in domains with different superconducting phases if Josephson phases of the structure parts are different, and vice versa. This process can be controlled by current injection through the sFS or sFNS parts. The domain formation significantly changes the critical current of the MJJ. This MJJ provides fast read and write operations with no need for an external magnetic field. Still, fabrication of compact Josephson junctions having the inhomogeneous weak-link region with reproducible characteristics is a difficult technological task.

#### OST-MRAM

The next considered type of cryogenic memory is the hybrid approach combining superconducting control circuits with spintronics memory devices. Here, due to spin-based interactions between atoms in the crystal lattice and electrons, orientations of ferromagnets magnetization can determine the amount of current flow. And vice versa, a spin-polarized current can affect orientations of the magnetizations. The latter effect is the so-called “spin transfer torque” (STT). It was suggested as a control mechanism for magnetic memory [170–172]. However, high speed and low energy of write operation can not be provided with conventional spin-valve topology with collinear orientations of ferromagnets magnetizations [173].

**Figure 16:**
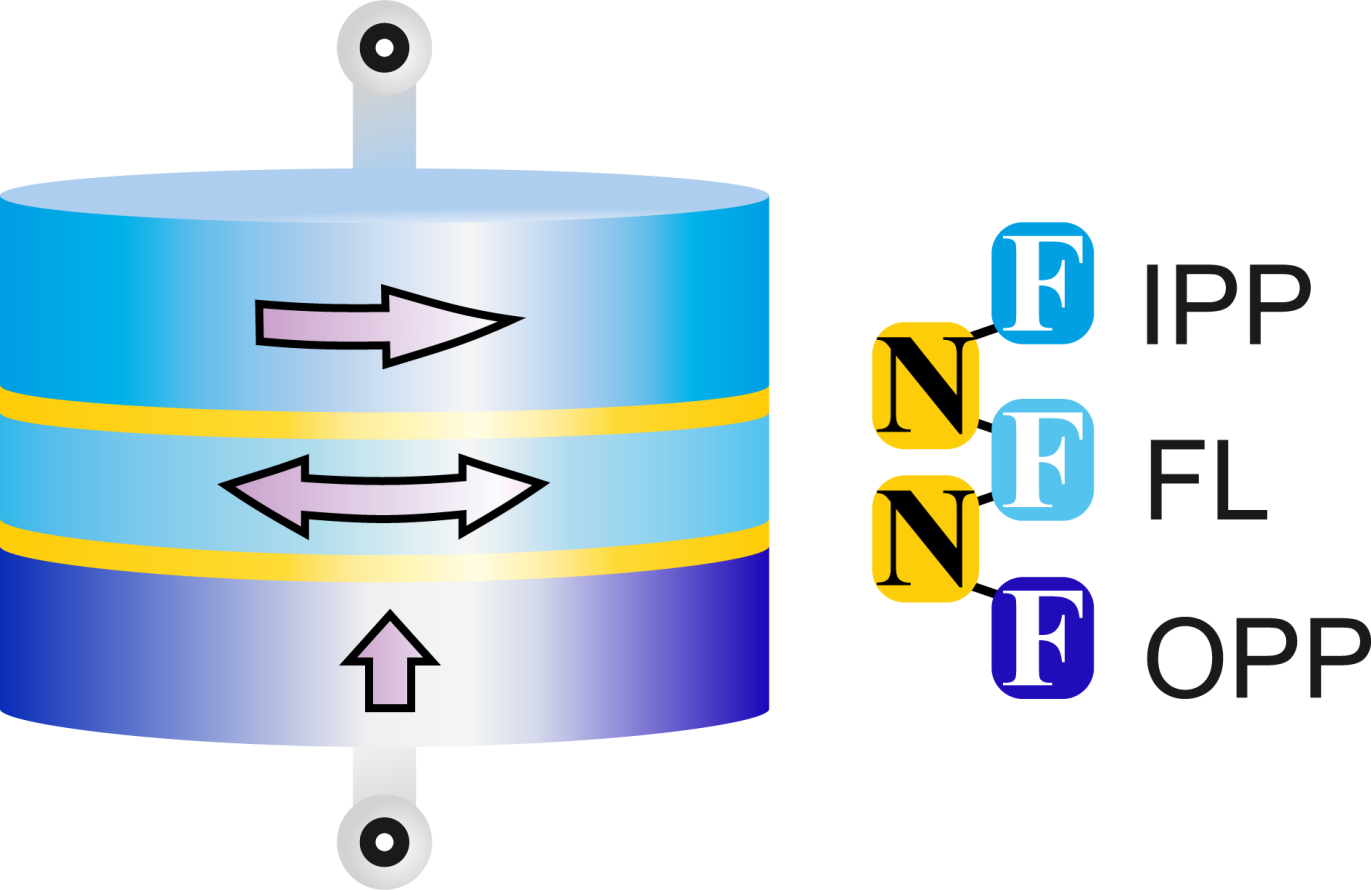
Sketch of an orthogonal spin transfer (OST) device. Arrows show magnetization directions in the device layers.

Orthogonal spin transfer (OST) devices allow one to overcome the difficulties. This structure consists of an out-of-plane ferromagnetic polarizer (OPP), a free F-layer (FL), and a fixed F in-plane polarizer/analyzer (IPP), see Figure 16. A “write” current pulse passing through the OPP leads to an STT effect in the FL, which acts to lift its magnetization out of plane. The magnetization is then rotated about the out-of-plane axis, according to the Landau–Lifshitz–Gilbert equation. A current pulse applied to IPP reads out collinear or anti-collinear magnetizations of the in-plane magnetized F-layers.

It is possible to obtain the necessary 180° magnetization reversal with the proper choice of the write pulse amplitude and duration. Quasi-static and dynamic switching characteristics of OST devices have been analyzed at cryogenic temperatures: switching between parallel and anti-parallel spin-valve states has been demonstrated for milliampere current pulses of sub-nanosecond duration [173–175].

Clear advantages of the considered approach are the elimination of control lines for the magnetic field application, and the implementation of a fast magnetization reversal at the sub-nanosecond timescale. At the same time, problems like relatively low magnetoresistance, and the ones associated with a possible over-rotation of magnetization still prevent its practical application. The latter one can be overcome to some extend by involving both IPP and OPP polarizers into the FL switching process [173].

The application of the STT effect in some of the MJJ valves is of considerable interest. STT in voltage-biased superconducting magnetic nanopillars (SFNFS and SFSFS junctions) has been studied for both equilibrium and nonequilibrium cases [176–180]. However, rich dynamics resulting from an interplay of multiple Andreev reflections, spin mixing, spin filtering, spectral dynamics of the interface states, and the Josephson phase dynamics requires further research to evaluate the applicability of STT in superconducting memory structures.

#### Discussion

The lack of suitable cryogenic RAM is “… the main obstacle to the realization of high-performance computer systems and signal processors based on superconducting electronics” [181]. While JMRAM and OST-MRAM look as the most advanced approaches, they still require further improvement in a number of critically important areas. Progress in the described variety of device types with no clear winner is impossible without researches on new magnetic materials, such as PdFe, NiFe(Nb,Cu,Mo), Co/Ru/Co and [Co/Ni]*_n_*, and novel magnetization reversal mechanisms [182–184]. They can lead to development of new operation principles combining superconductivity and spintronics.

The inverse proximity effect at SF boundaries requires the use of quite thin (nanometer scale) magnetic layers. However, the characteristics of memory devices typically depend exponentially on the thickness of the F-layers and are significantly affected by interfacial roughness. This challenge can be met with further development of high-accuracy thin-film technological processes in modern fabrication technology.

A substantial part of circuit area, time delay and dissipated power in the memory matrix is more likely to be associated with address lines than with memory cells. This makes the optimization of intra-matrix interconnections and memory cell architecture of significant importance.

While we considered here only the most developed solutions for superconducting valves and memory elements, there are many other approaches to create nanosized controllable superconducting devices for applications in memory and logic. We can point out at our discretion: the nanoscale superconducting memory based on kinetic inductance [185], and the superconducting quantum interference proximity transistor [186]. These concepts could bring novel ideas into the nanoscale design of superconducting circuits.

## Conclusion

In conclusion, we discussed different superconductor logics providing fast (5–50 GHz) and energy-efficient (10^−19^–10^−20^ J per bit) operation of circuits in non-adiabatic and adiabatic regimes. The latter allows for the implementation of the most energy-efficient physically and logically reversible computations with no limit for minimum energy dissipation per logic operation. Possibilities to combine the schemes based on different logics as well as the use of different (e.g., superconductor and semiconductor) technologies in a single device design are presented.

The physical principles underlying the operation of superconductor circuits provide a possibility for the development of devices based on unconventional computational paradigms. This could be the basis for a cryogenic cross-platform supercomputer, where each task can be executed in the most effective way. In our opinion, the development of superconductor circuits performing non-classical computations like cellular automata, artificial neural networks, adiabatic, reversible, and quantum computing is indispensable to obtain all the benefits of superconductor technology.

Low integration density, and hence low functional complexity of the devices, is identified as the major problem of the considered technology. This issue can be addressed with further miniaturization of basic elements and modernization of cell libraries, including the introduction of novel devices such as the ones based on nanowires or magnetic Josephson junctions.

The problem of low integration density is especially acute in RAM design. We considered here four different approaches to cryogenic RAM development with no clear winner. Progress in this area now implies the elaboration of new operation principles based on a synergy of different physical phenomena such as superconductivity and magnetism, and the appearance of novel effects, as for example, triplet spin valve memory effect [142] or superconducting control of the magnetic state [157]. Proposed concepts of new controllable devices could eventually change the face of superconductor technology making it universal platform of future high-performance computing.
